# Technical Complications during Veno-Venous Extracorporeal Membrane Oxygenation and Their Relevance Predicting a System-Exchange – Retrospective Analysis of 265 Cases

**DOI:** 10.1371/journal.pone.0112316

**Published:** 2014-12-02

**Authors:** Matthias Lubnow, Alois Philipp, Maik Foltan, Tone Bull Enger, Dirk Lunz, Thomas Bein, Assad Haneya, Christof Schmid, Günter Riegger, Thomas Müller, Karla Lehle

**Affiliations:** 1 Department of Cardiothoracic Surgery, University Medical Center Regensburg, Franz-Josef-Strauss-Allee 11, 93042 Regensburg, Germany; 2 Department of Internal Medicine II, University Medical Center Regensburg, Franz-Josef-Strauss-Allee 11, 93042 Regensburg, Germany; 3 Department of Anesthesiology, University Medical Center Regensburg, Franz-Josef-Strauss-Allee 11, 93042 Regensburg, Germany; Medizinische Hochschule Hannover, Germany

## Abstract

**Objectives:**

Technical complications are a known hazard in veno-venous extracorporeal membrane oxygenation (vvECMO). Identifying these complications and predictive factors indicating a developing system-exchange was the goal of the study.

**Methods:**

Retrospective study on prospectively collected data of technical complications including 265 adult patients (Regensburg ECMO Registry, 2009-2013) with acute respiratory failure treated with vvECMO. Alterations in blood flow resistance, gas transfer capability, hemolysis, coagulation and hemostasis parameters were evaluated in conjunction with a system-exchange in all patients with at least one exchange (n = 83).

**Results:**

Values presented as median (interquartile range). Patient age was 50(36–60) years, the SOFA score 11(8–14.3) and the Murray lung injury Score 3.33(3.3–3.7). Cumulative ECMO support time 3411 days, 9(6–15) days per patient. Mechanical failure of the blood pump (n = 5), MO (n = 2) or cannula (n = 1) accounted for 10% of the exchanges. Acute clot formation within the pump head (visible clots, increase in plasma free hemoglobin (frHb), serum lactate dehydrogenase (LDH), n = 13) and MO (increase in pressure drop across the MO, n = 16) required an urgent system-exchange, of which nearly 50% could be foreseen by measuring the parameters mentioned below. Reasons for an elective system-exchange were worsening of gas transfer capability (n = 10) and device-related coagulation disorders (n = 32), either local fibrinolysis in the MO due to clot formation (increased D-dimers [DD]), decreased platelet count; n = 24), or device-induced hyperfibrinolysis (increased DD, decreased fibrinogen [FG], decreased platelet count, diffuse bleeding tendency; n = 8), which could be reversed after system-exchange. Four MOs were exchanged due to suspicion of infection.

**Conclusions:**

The majority of ECMO system-exchanges could be predicted by regular inspection of the complete ECMO circuit, evaluation of gas exchange, pressure drop across the MO and laboratory parameters (DD, FG, platelets, LDH, frHb). These parameters should be monitored in the daily routine to reduce the risk of unexpected ECMO failure.

## Introduction

Over the last 40 years, extracorporeal membrane oxygenation (ECMO) has been used to support adult patients with respiratory or cardiac failure who are unlikely to survive conventional mechanical ventilation [1]. Pivotal progress in extracorporeal technology, encouraging results of the efficacy and economic assessment of ECMO for severe adult respiratory failure (CESAR) trial [2], [3] and good outcomes of ECMO patients during the recent H1N1 influenza pandemic [4], [5] have contributed to a resurgence of interest in ECMO therapy [6], [7]. Improvements in ECMO circuitry, pump and oxygenator technology permit safer perfusion for longer periods of time. Respective clinical benefit of veno-venous (vv) ECMO support was reviewed by Combes and coworkers [8]. However, none of the recently published systematic reviews and pooled analyses of retrospective studies focused on the development of technical complications while on ECMO [9].

This is the first experience report by a single ECMO center to evaluate early technical complications on ECMO, which might be relevant for a system-exchange.

## Materials and Methods

The Regensburg ECMO database was queried for all consecutive patients on vvECMO (January 2009 to December 2013, n = 265). Only patients with at least one system-exchange and an ECMO support duration of more than 48 hours were included. Among patients needing multiple ECMO treatments, only the first was included. Prospectively collected physical and laboratory parameters allowed a retrospective analysis of the reasons for a system-exchange in 83 patients. Ethical approval for publication of this retrospective analysis and need for informed consent was waived by the Ethics Committee of the University of Regensburg, as all devices are approved for clinical use, no personalized data and only routine laboratory parameters were used. Patient characteristics are shown in Table 1. Indications for vvECMO are shown in Table 1 and in file S4.

**Table 1:** Patient data and characteristics before ECMO.

	All	No exchange	System-exchange	p-value
Patients (n)	265	182	83	
Age (years)	50 (36–60)	50 (37–61)	50 (36–60)	0.844
Females (n; %)	90; 34	59; 32	31; 37	0.518
BMI (kg * m^-2^)	27.8 (24.7–33.1)	28.1 (24.8–32.9)	27.5 (23.9–34.2)	0.545
Ventilation (days)	1.0 (1.0–6.0)	1.0 (1.0–5.3)	2.0 (1.0–6.5)	0.431
SOFA score	11.0 (8.0–14.3)	12.0 (8.0–15.0)	11.0 (8.0–13.0)	0.067
LIS	3.33 (3.33–3.67)	3.33 (3.33–3.67)	3.33 (3.33–3.67)	0.660
ARF (n; %)	60; 22	55; 30	5; 6	≤0.001
NE (µg/kg/min)	0.26 (0.10–0.51)	0.27 (0.10–0.51)	0.22 (0.08–0.53)	0.425
PaO_2_/FiO_2_ (mmHg)	65 (52–85)	65 (51–84)	69 (52–86)	0.554
PaCO_2_ (mmHg)	64 (51–80)	63 (50–79)	64 (54–88)	0.252
apH	7.24 (7.15–7.34)	7.23 (7.13–7.33)	7.24 (7.15–7.35)	0.453
TV (ml)	470 (396–562)	477 (399–565)	455 (358–560)	0.447
TV/kg pred. BW (ml/kg)	5.6 (4.2–6.7)	5.6 (4.4–6.7)	5.3 (3.8–6.7)	0.413
Minute ventilation (l/min)	10.6 (8.3–12.6)	11.0 (8.6–12.6)	10.5 (7.9–12.6)	0.517
PIP (cm H_2_O)	35 (30–39)	35 (30–39)	35 (30–40)	0.813
PEEP (cm H_2_O)	15 (13–18)	15 (13–18)	15 (13–18)	0.505
ECMO indication §				0.001
1 (n; %)	150; 57	92; 51	58; 70	
2 (n; %)	53; 20	45; 25	8; 10	
3 (n; %)	33; 12	28; 15	5; 6	
4 (n; %)	29; 11	17; 9	12; 14	

Data are presented as median (interquartile range) except for females and acute renal failure. SOFA, Sequential Organ Failure Assessment; LIS, Murray lung injury score; apH, arterial pH value; PaCO_2_, partial pressure of arterial carbon dioxide; PaO_2_/FiO_2_, ratio of partial pressure of arterial oxygen and fraction of inspired oxygen; PIP, peek inspiratory pressure; PEEP, positive end-expiratory pressure; TV, tidal volume; BMI, body mass index; ARF, acute renal failure; NE, Norepinephrine.

§ ECMO indications: 1, primary lung failure (bacterial, viral, fungal, aspiration pneumonia and H1N1 infection); 2, sepsis with secondary lung failure; 3, trauma with ARDS; 4, other pathologies (eg. pulmonary fibrosis, pulmonary hypertension, pulmonary emboli, extensive bronchiectasis, pulmonary bleeding, tracheal laceration).

### Standard treatment for ECMO patients

When ECMO is initiated, mechanical ventilation is reduced based on the blood gases, aiming for a fraction of inspired oxygen (FiO_2_) of <60%, peak inspiratory pressure <26–28 cmH_2_O, and positive end expiratory pressure (PEEP) aiming for lung recruitment according to underlying disease usually >10 cmH_2_O [10]. The tidal volume (TV) is kept between 3–6 ml/kg ideal bodyweight according to peak pressure and the proportion of arreated lung tissue as indicated on a computerized tomography (CT) scan. The respiratory rate (RR) is kept between 10–30/min. Arterial blood gases (under stable conditions) are drawn every 4–6 hours. The ECMO blood flow, FiO_2_ and PEEP are adjusted to maintain an arterial oxygen saturation of >90%. The sweep gas flow rate, TV and RR are adjusted according to the arterial partial pressure of carbon dioxide (PaCO_2_), aiming for a normal pH. As sweep gas in vvECMO, 100% O_2_ is used to utilize maximal O_2_ transfer capacity. Hemoglobin is kept ≥8–9 g/dl and platelet count >20/nl. The anticoagulation protocol was based on intravenous heparin application. In patients without an elevated bleeding risk, an activated partial thromboplastin time (aPTT) of 60 seconds was maintained, and in patients with a moderately increased bleeding risk an aPTT of 40 to 50 seconds was used. In trauma patients with severe or multiple bone fractures, or patients with bleeding diathesis (thrombocytopenia, disseminated intravascular coagulopathy [DIC], etc.), ECMO support was started without administering heparin for up to 7 days, relying on the heparin or phosphorylcholin coating of the system. APTT was controlled and readjusted at least twice daily (details see [10], [11]). The following lab values were drawn daily and as necessary: ECMO blood gases, complete blood count, coagulation including D-Dimers (DD), plasma free hemoglobin (frHb), basic metabolic panel, magnesium, ionized calcium, and phosphorous. Respiratory parameters included peak inspiratory pressure (PIP), PEEP, TV, minute ventilation and RR. In addition, blood products used, plus dosages of norepinephrine and heparin were documented. A chest radiograph or CT scan was performed when indicated.

### ECMO systems and measurements of function

Miniaturized ECMO systems consist of an electrical drive unit, a blood pump, MO and tubing. Six different ECMO-systems were used: (PLS-System [n = 112], Cardiohelp HLS-set advanced [n = 51], Quadrox-ID Pediatric oxygenator + Rotaflow pump [n = 4], Maquet Cardiopulmonary, Hirrlingen, Germany; Hilite7000LT oxygenator + DP3 pump [n = 47], Medos Medizintechnik, Stolberg, Germany; iLA-activve-system [n = 15], Novalung, Heilbronn, Germany; ECC.05 system [n = 36], Sorin Group, Modena, Italy). The main differences between the ECMO systems used were the types of blood pumps and biocompatible coatings (PLS-system, Rotaflow  =  centrifugal pump, Heparin based Bioline-coating; Cardiohelp-system, Rotassist  =  integrated centrifugal pump, Heparin based Bioline-coating; Hilite-system, Deltastream, DP3  =  diagonal pump, Heparin based Rheoparin-coating; ECC.05-system, Revolution  =  integrated centrifugal pump, Phosphorylcholin-coating), as well as the design of the MO (hollow fiber packing density, winding technique, gas exchange area). The gas exchange membrane is identical for all systems used and was made of polymethylpentene (PMP) (Membrana, Wuppertal, Germany). In case of technical complications the whole ECMO circuit (tubing, MO, blood pump), except for the cannulas, was exchanged. Cannulas were seleced independent of the type of ECMO system (single-lumen cannula (n = 191, Maquet), single dual-lumen cannula – AvalonElite bi-caval dual lumen catheter (n = 55, Maquet), single dual-lumen cannula - NovaportTwin (n = 19, Novalung). The systems and cannulas most commonly used were described earlier [10]. The extracorporeal support was initiated with a blood flow of approximately 1.5–2 l/min and a sweep gas flow of 1–3 l/min. Thereafter, blood flow was adjusted according to arterial oxygen saturation (SaO_2_), and gas flow according to PaCO_2_ and pH level. Transmembrane pressure drop was defined as the difference between pressure at the inlet and outlet of the MO (dpMO = pMO_in_ – pMO_out_) [12]. Blood gases at the device inlet and outlet were also analyzed (Radiometer ABL800 FLEX, Bronshoj, Denmark).

### Technical complications demanding an acute system-exchange

Mechanical failure was defined as leakage at the MO, connectors or pump head.

Acute clot formation usually affects the MO or the pump head [13]. Major clots within the MO (acute oxygenator thrombosis, AOT) caused an acute increase in the dpMO, accompanied by a decrease in blood flow at the same pump speed [12]. Pump head thrombosis (PHT) can be indicated by a sudden sound change of the blood pump, technically-induced hemolysis [14], [15], [16] and/or a decrease in platelet count. Differential diagnosis resulting in elevated frHb, such as powerful aspiration of the blood sample, excessive suction due to cannula malposition (mostly combined with high negative inflow pressures, inadequate high pump speed proportionately to blood flow), continuous renal replacement therapy, thrombosis or larger hematomas have to be ruled out.

The approach to an acute system-exchange is detailed in file S4.

### Technical complications demanding an elective system-exchange

#### Worsening of gas exchange capability

Prolongation of ECMO support and the existence of a coagulation disorder promote the progression of clot formation within the circuit (especially within the MO). Thrombotic, fibrin or cellular deposits on the surface of the gas exchange membranes worsen the gas transfer capability of the MO [12], ultimately requiring an elective system-exchange.

In the present study, worsened gas exchange capability was defined as follows:

1. A significant decrease in the partial pressure of O_2_ in the blood at the outlet of the MO (pO_2 post_MO) of more than 50% compared to the initial value and
2. An elevation of the partial pressure of CO_2_ in the blood at the outlet of the MO (pCO_2 post_MO) over 40 mmHg at high gas flow rates (≥10 l/min)

#### Device-induced coagulation disorder

Coagulation was monitored by measuring activated prothrombin time (aPT), aPTT, fibrinogen (FG), antithrombin III (AT III), DD and platelet count. Markers for a device-induced coagulation disorder were an otherwise unexplainable (e.g. DIC, thrombosis, pulmonary embolism, trauma, surgery) increase in the levels of DD from <10 mg/dl to 25–35 mg/dl [17] (fibrinolysis in the MO), or an otherwise unexplainable increase in DDs and a decrease in FG concentration (<200 mg/dl) (device-induced hyperfibrinolysis) with subsequent improvement after exchange. Thus, device-induced coagulation disorder is presumptive until the exchange. These markers were mostly accompanied by a decrease in platelet count and a diffuse unaccountable bleeding tendency before system-exchange, which normally reversed after exchange.

#### Suspected infection of the ECMO circuit

Another reason for an elective system-exchange was suspicion of circuit infection [18], [19], which was based on clinical observation, positive blood cultures, increase in inflammatory parameters (C-reactive protein [CRP], leukocytes, fever) without attributable focus and a diffuse, unaccountable bleeding diathesis. No routine infection surveillance (e.g. blood cultures at defined days) was performed.

### Statistics

Data are expressed as median (25–75 percentile) and were analyzed with the Wilcoxon-signed-rank test (SigmaStat 3.1, Systat Software, San Jose, CA, USA). Data were analyzed for normality (Kolmogorov-Smirnov test) and homogeneity of variance (Levene). Intergroup differences were compared using the Friedman test for repeated measures analysis of variance (ANOVA) by ranks, with post-hoc Dunn's Method for multiple comparison versus control (time of system-exchange). P-values ≤0.05 indicated a significant difference.

## Results

### Study population

Indications for vvECMO were primary lung failure in 68% and secondary lung failure in 32%. 46 patients had a platelet count of <80/nl before inclusion, and 67 patients displayed signs of DIC (aPTT of >60 sec, reduced aPT, AT III, FG and elevated DD levels). All but 25 patients depended on norepinephrine, and 22% presented with acute renal failure, defined as the need for renal replacement therapy. Patient characteristics before ECMO are summarized in Table 1.

**Figure 1:**
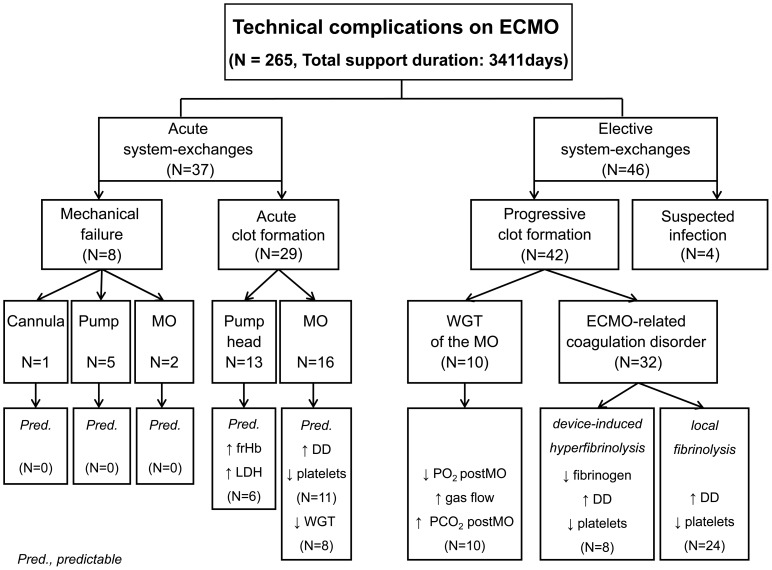
Retrospective analysis of reasons for acute and elective system-exchange during vvECMO. Acute exchanges were all events that required an immediate intervention of perfusionists. Mechanical failure was defined as leakage at the MO, pump head problems, dysfunction of the pumpdrive, cannula or circuit rupture. Acute clot formation within the MO caused a severe increase in the pressure drop across the MO (dpMO) followed by a decrease in blood flow, while acute clot formation in the pump head was indicated by a dramatic increase in plasma free hemoglobin concentration. Suspected infection based on clinical observations (see text). Progressive clot formation was observed in almost all remaining MOs, which caused a worsened gas exchange capability of the MO (decrease in pO_2 post_MO, increase in pCO_2 post_MO, increase in sweep gas flow) and an alteration in coagulation parameters (increase in D-dimer levels, decrease in fibrinogen levels and decrease in platelet counts).

Eighty-three patients (31%) required one or more system-exchanges due to technical complications (Figure 1). Patient data and characteristics before ECMO of both groups (Table 1) were comparable, except that patients with a system-exchange had less renal failure pre-ECMO (p≤0.001), the SOFA score tended to be lower (p = 0.067), and the reason for ARDS was more often primary lung failure and less frequently trauma-related lung failure (p = 0.001). In both groups, respiratory (e.g. PEEP, PIP, TV, minute ventilation, RR) or coagulation parameters (aPTT, aPT, FG, DD, AT III, LDH, platelets) and dosages of heparin or norepinephrine pre-ECMO were comparable (see file S1). Patients requiring at least one system-exchange had a longer ECMO support duration (p≤0.001), total ventilation time (p≤0.001), residence time in the intensive care unit (ICU) (p≤0.001) and a higher risk of renal failure requiring renal replacement therapy during ECMO (p = 0.028) (Table 2). Despite prolongation of ECMO support and implantation of 2 to 5 MOs, there was no difference in mortality between both groups (p = 0.113) (Table 2). Comparing patients requiring an acute (37/83) and elective system-exchange (46/83), those requiring an acute system-exchange presented with more severe respiratory acidosis (Table 3) and had a shorter working time of the first MO (p = 0.023). Patients requiring an elective system-exchange presented with significantly higher CRP values before ECMO therapy, tended to have longer durations of ECMO support (p = 0.095), more often required several MOs (p = 0.079) and were ventilated longer (p = 0.045). However, during and after ECMO support, the consumption of blood products and outcome parameters did not differ significantly between acute and elective exchanges, and in particular mortality was not increased (Table 4). Furthermore, there were no differences with respect to anticoagulation, or coagulatory and inflammatory parameters before system-exchange (see file S2).

**Table 2:** Patient data during and after ECMO support.

	All	No exchange	System-exchange	p-value
Patients (n)	265	182	83	
Stay in ICU (days)	25 (16 – 38)	21 (14–34)	34 (24–46)	≤0.001
Intubation (days)	20 (13–32)	16 (11–26)	30 (20–43)	≤0.001
ARF (n; %)	51; 19	28; 15	23; 28	0.028
Amount of MOs		1	2.0 (2.0–3.0)	≤0.001
Cumulative support time (days)	3411	1538	1873	
Support time 1^st^ MO (days)		8 (6–10)	9 (6–12)	0.073
ECMO duration (days)	9 (6–15)	8 (6–10)	20 (14–27)	≤0.001
Successful weaning (n; %)	200; 75	143; 79	57; 69	0.113
Died on ECMO (n; %)	65; 25	39; 21	26; 31	0.113
RBC/days ECMO	0.25 (0.00–0.66)	0.20 (0.00–0.67)	0.31 (0.15–0.57)	0.180
FFP/days ECMO	0.00 (0.00–0.25)	0.00 (0.00–0.20)	0.00 (0.00–0.26)	0.463
PC/days ECMO	0.00 (0.00–0.00)	0.00 (0.00–0.00)	0.00 (0.00–0.05)	0.019

Data are presented as median (interquartile range) except for successful weaning and acute renal failure on ECMO. MO, membrane oxygenator; ICU, intensive care unit; RBC, red blood cells; FFP, fresh frozen plasma (1 FFP contains 230 ml plasma); PC, platelet concentrate (1 PC contains 250 ml and 2–4×10^11^ thrombocytes); ARF, acute renal failure.

**Table 3:** Patient data and characteristics before ECMO comparing acute vs. elective system-exchange.

	Acute	Elective	p-value
Patients (n)	37	46	
Age (years)	50 (36–58)	50 (36–61)	0.657
Females (n; %)	12; 32	19; 41	0.547
BMI (kg * m^−2^)	26.8 (23.9–34.6)	27.7 (23.9–33.1)	0.887
Ventilation (days)	1.0 (1.0–4.0)	2.0 (1.0–9.0)	0.350
SOFA score	11.0 (7.8–13.3)	11.0 (8.0–13.0)	0.652
LIS	3.33 (3.00–3.67)	3.67 (3.33–3.67)	0.196
ARF (n; %)	1; 2.7	4; 8.7	0.375
NE (µg/kg/min)	0.24 (0.07–0.51)	0.21 (0.08–0.56)	0.812
PaO_2_/FiO_2_ (mmHg)	70 (52–89)	65 (54–80)	0.677
PaCO_2_ (mmHg)	71 (57–103)	64 (53–73)	0.039
apH	7.20 (7.12–7.27)	7.29 (7.19–7.37)	0.005
TV (ml)	450 (350–560)	477 (390–565)	0.495
TV/kg pred. BM (ml)	5.01 (3.76–6.17)	5.68 (3.87–7.80)	0.205
Minute ventilation (l/min)	9.0 (6.4–12.2)	11.0 (9.2–13.4)	0.025
PIP (cm H_2_O)	33 (30–40)	35 (31–38)	0.582
PEEP (cm H_2_O)	14 (12–19)	15 (13–18)	0.529
Platelets pre (/nl)	178 (100–371)	206 (103–283)	0.647
Hemoglobin (g/dl)	10.8 (9.1–13.0)	9.9 (8.6–11.5)	0.110
CRP pre (mg/l)	107 (36–206)	177 (108–250)	0.041
Leukocytes (/nl)	11.8 (9.0–19.9)	12.9 (7.5–21.2)	0.927
ECMO indication §			0.910
1 (n; %)	26; 70	32; 70	
2 (n; %)	2; 5	6; 13	
3 (n; %)	3; 8	2; 4	
4 (n; %)	6; 16	6; 13	

Data are presented as median (interquartile range) except for females, acute renal failure. SOFA, Sequential Organ Failure Assessment; LIS, Murray lung injury score; apH, arterial pH value; PaCO_2_, partial pressure of arterial carbon dioxide; PaO_2_/FiO_2_, ratio of partial pressure arterial oxygen and fraction of inspired oxygen; PIP, peek inspiratory pressure; PEEP, positive end-expiratory pressure; TV, tidal volume; BMI, body mass index; ARF, acute renal failure; NE, norepinephrine; CRP, c reactive protein.

§ ECMO indications: 1, primary lung failure (bacterial, viral, fungal, aspiration pneumonia and H1N1 infection); 2, sepsis with secondary lung failure; 3, trauma with ARDS; 4, other pathologies (eg. pulmonary fibrosis, pulmonary hypertension, pulmonary emboli, extensive bronchiectasis, pulmonary bleeding, tracheal laceration).

**Table 4:** Patient data during and after ECMO support of system-exchange patients.

	Acute	Elective	p-value
Patients (n)	37	46	
Stay in ICU (days)	32 (19–43)	38 (24–47)	0.106
Intubation (days)	23 (16–43)	32 (23–45)	0.045
ARF on ECMO (n; %)	10; 27	13; 33	0.903
Amount of MOs	2.0 (2.0–2.0)	2.0 (2.0–3.0)	0.079
Cumulative support time (days)	744	733	
Support time 1^st^ MO (days)	7.0 (5.0–10.3)	9.5 (7.0–13.0)	0.023
ECMO duration (days)	17.0 (11.8–26.0)	22.0 (15.0–30.0)	0.095
Successful weaning (n; %)	28; 76	29; 63	-
Died on ECMO (n; %)	9; 17	17; 29	0.320
RBC/days ECMO	0.24 (0.00–0.72)	0.32 (0.25–0.56)	0.147
FFP/days ECMO	0.00 (0.00–0.01)	0.00 (0.00–0.33)	0.054
PC/days ECMO	0.00 (0.00–0.01)	0.00 (0.00–0.06)	0.488

Data are presented as median (interquartile range) except for successful weaning and acute renal failure on ECMO. MO, membrane oxygenator; ICU, intensive care unit; RBC, red blood cells; FFP, fresh frozen plasma (1 FFP contains 230 ml plasma); PC, platelet concentrate (1 PC contains 250 ml and 2–4×10^11^ thrombocytes); ARF, acute renal failure.

### Technical complications on ECMO

Figure 1 provides an overview of relevant technical complications on ECMO.

### Acute system-exchanges

#### Mechanical failure

Eight patients required an urgent system-exchange due to mechanical failure. A blood leakage at the gas outlet port of a Medos-MO, or at the inlet connector of a PLS-MO made system-exchange inevitable. Failure of blood pumps was caused by electrical failure of the pump drive bridged by use of a hand crank (n = 3), material defect of the plastic housing (n = 1), or imbalance of the propeller within the pump head that produced abnormal noise (n = 1). In one patient an air leakage of a bicaval dual lumen cannula (AvalonElite, Maquet) was documented after 30 days. Intense head movements may have caused the partial cannula rupture. Urgent clamping and a system stop averted further sequelae. There was no circuit or tubing rupture.

#### Pump head thrombosis

**Figure 2:**
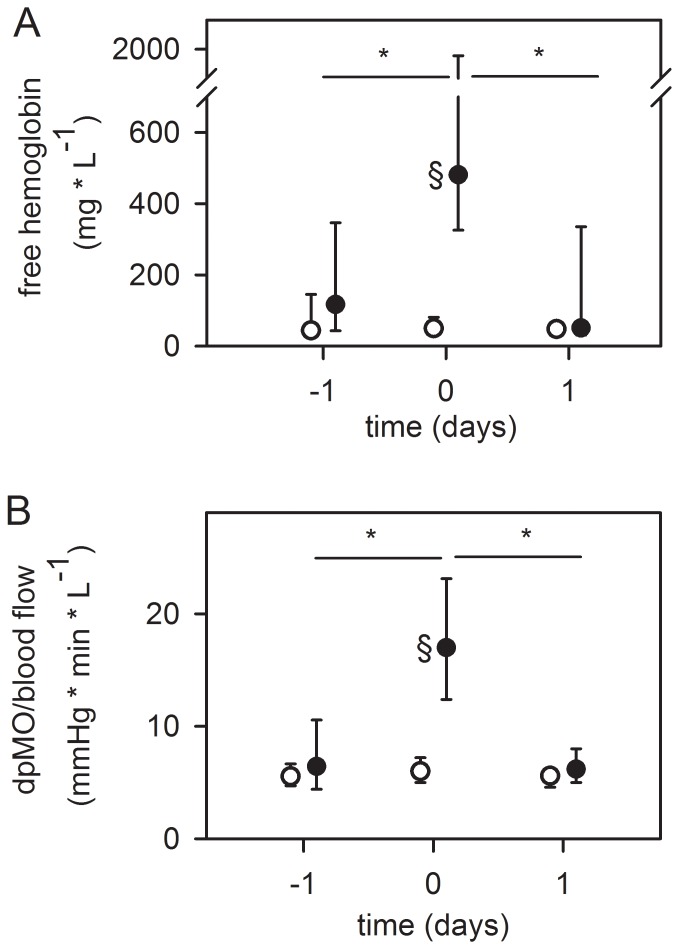
Pump head thrombosis (top, A) and acute oxygenator thrombosis (bottom, B). (A) Alterations in plasma free hemoglobin concentration in patients with pump head thrombosis one day before, at system-exchange and one day thereafter (n = 13, black dots). (B) Alterations in pressure drop across the oxygenator (dpMO) normalized by blood flow in patients with acute oxygenator thrombosis one day before, at system-exchange and one day thereafter (n = 16, black dots). Circles in both graphs: Patients without system exchange and a comparable support time of the MO (≥12 days, n = 36) were used as control group. Since the system-exchange was necessary after 9 (6–12) days (Table 2), values at day 9 after starting ECMO therapy were set as “day 0” and days −1 and 1 depicted accordingly. Data are presented as median and interquartile range. *, p≤0.05 compared to day 0 (refers to black dots); §, p≤0.001 compared to patients without system exchange (circles).

PHT (n = 13) was indicated by a significant increase in the levels of frHb (10 [4]–[15] -fold, p≤0.001) before the exchange, and a significant decrease thereafter (6 [4]–[18] -fold, p≤0.001) (Figure 2). Clots within each pump head were documented after removal. In 6/13 patients, increased frHb levels could already be seen one day before PHT (day 2 before PHT, 55 [49–60] mg/dl; day 1 before PHT, 143 [117–266] mg/dl, p = 0.031). Patients without system-exchange showed significantly less frHb after a comparable support time.

#### Acute oxygenator thrombosis

AOT was verified by a significant increase in the ratio of dpMO/blood flow before exchange (2.6 [2.1–4.0] -fold, p≤0.001), followed by a subsequent drop after exchange (3.2 [2.0–5.2] -fold, p≤0.001) (Figure 2). In all other patients with acute system-exchanges, no increase in dpMO/blood flow was noted. Only in 1 patient with an elective system-exchange (due to suspicion of infection) was an increase in dpMO/blood flow by a factor of 3.1 seen. This is probably a result of an exchange before an AOT happened due to progressive thrombosis obstructing main parts of the oxygenator.

In 11/16 patients with AOT, DD levels were elevated before AOT (31 [23–35] mg/dl) and declined within 2 days after the system-exchange (13 [6–31] mg/dl, p = 0.016). The other 5/16 patients had a sudden AOT missed by routine testing of DD. There was only a trend towards a decreased platelet count (2 days before AOT, 161 [76–300]/nl; day of AOT, 145 [74–286]/nl, not significant), which improved in 6/11 patients after exchange. In addition, in 8/11 patients, the gas transfer capability of the MOs decreased before AOT (53 [45–59]% decrease in pO_2 post_MO; higher levels of CO_2 post_MO (40 [32–48] mmHg) at high gas flow rates of 10 [9]–[11] l/min). Additionally, 2 of 4 patients receiving activated coagulation factor seven while on ECMO developed an AOT requiring a system-exchange.

### Elective system-exchanges

#### Suspected circuit infection

In four patients (5%) the system was exchanged due to suspected infection. In one patient with Landouzy sepsis, mycobacteria were identified microscopically in the MO after exchange. Another MO was colonized with Enterococcus spp. verified after ex-vivo long-term incubation with culture medium. Clinical course and septic history of two patients demanded an exchange due to suspected circuit infection. These patients developed a diffuse, unaccountable bleeding diathesis after long-term ECMO support (15–18 days) that ceased after system-exchange. Furthermore, elevated inflammatory parameters tended to decrease after system-exchange (day of/after exchange; leukocytes: 15.6 (9.3–17.2)/nl/14.6 (8.2–14.8)/nl; CRP: 147 (109–236) mg/dl/95 (92–110) mg/dl). In one patient norepinephrine dosage increased before system-exchange (5-fold; on day of exchange, 0.35 µg/kg/min) and dropped by a factor of 15 after MO replacement.

#### Progressive clot formation and worsening of gas transfer

Larger clots could be detected by visual control. Additionally, daily monitoring of gas transfer data, coagulation and hemolysis parameters allowed early identification of MOs with progressive clot formation and worsened gas transfer efficiency (n = 42, 51%).

**Figure 3:**
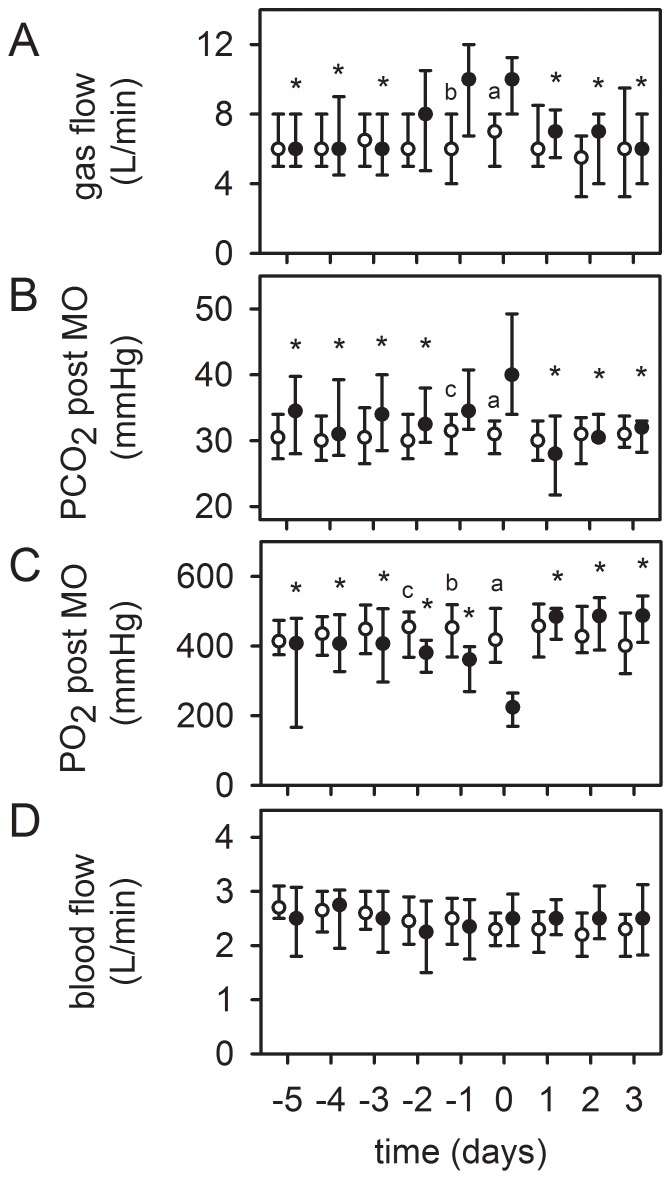
Worsened gas exchange capability as a reason for an elective system-exchange. Timeline of respective values before and after sytem-exchange, “day 0” =  system-exchange. Patients with system-exchange (black dots, n = 10). Despite an increase in gas flow rate (100% O_2_) (top, A), the partial pressure of CO_2_ at the outlet of the MO (pCO_2 post_MO) increased significantly (>40 mmHg) (middle, B). The oxygenation capability (pO_2 post_MO) decreased (middle, C). ECMO blood flow remained unchanged (bottom, D). After system-exchange, gas exchange data improved significantly. Circles in all graphs: Patients without system-exchange and a comparable support time of the MO (≥12 days, n = 36) were used as control group. Since the system-exchange was necessary after 9 (6–12) days (Table 2), values at day 9 after starting ECMO therapy were set as “day 0” and depicted accordingly. Data are presented as median and interquartile range. *, p≤0.05 compared to day 0 (refers to black dots). a, p≤0.001; b, p≤0.01; c, p≤0.05 compared to patients without system-exchange (circles).

In ten patients worsening of gas transfer capability of the MO was the reason for a system-exchange. Despite an increase in gas flow rate, pCO_2 post_MO increased significantly (>40 mmHg), while pO_2 post_MO was reduced, opposed to patients (n = 36) without system-exchange after a comparable support time (Figure 3).

The ventilatory aggressiveness (PIP, TV, minute ventilation, FiO_2_) rose slightly visually, without reaching statistical significance (see file S3). Immediately after the exchange, gas transfer data improved significantly and ventilatory aggressiveness decreased visually. In half of the patients, worsened gas exchange was accompanied by an alteration of coagulation parameters. Levels of DD increased in 5 patients from 14 (6–34) to 34 (32–35) mg/dl. FG concentration decreased in one patient (253 to 98 mg/dl). Platelet count declined in 3 patients from 193 (88–261)/nl to 53 (42–82)/nL. Removal of the MO resulted in a reduction of DD levels (13 [5]–[16] mg/dl, not significant) and delayed increases in FG concentration and platelet count over the next 5 days (not significant). In addition, 6 patients showed an increase in frHb levels before system-exchange (61 [30–85] mg/dl to 136 [119–196] mg/dl) that declined thereafter.

#### Device-related coagulation disorder

**Figure 4:**
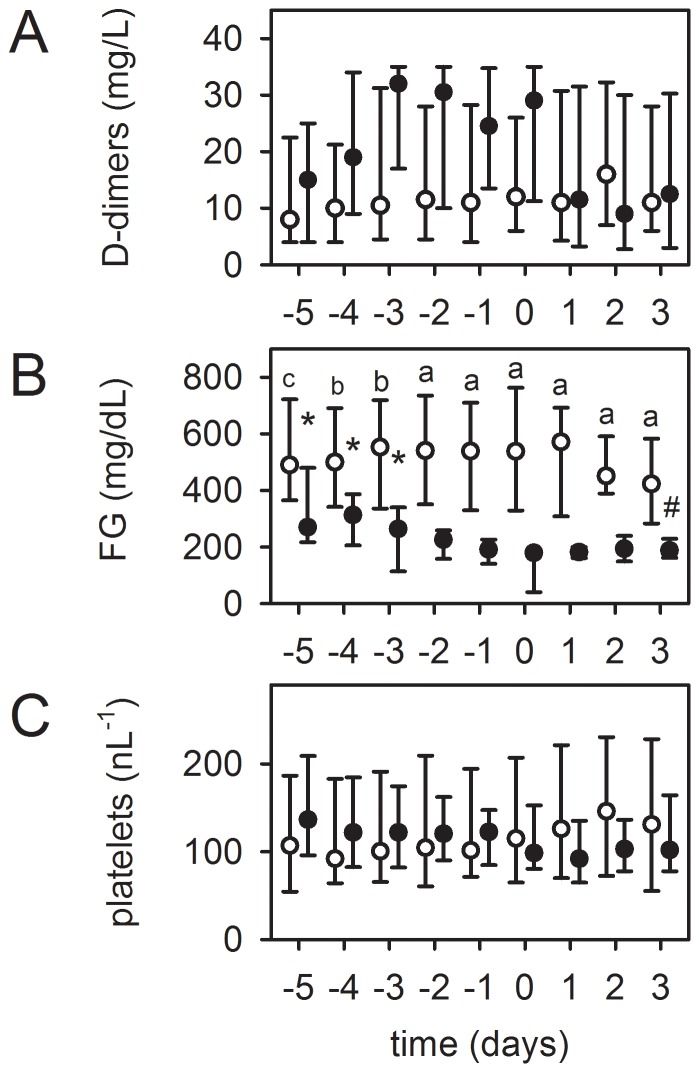
Device-induced hyperfibrinolysis as a reason for an elective system-exchange. Timeline of respective values before and after sytem-exchange, “day 0” =  system-exchange. Patients with system-exchange (black dots, n = 8): (B) Fibrinogen (FG) concentration decreased significantly below the normal value of 200 mg/dl, while (A) levels of D-dimers visually increased and (C) platelet count decreased (both not statistically significant). After system-exchange the coagulation disorder could be stopped. Circles: Patients without exchange and comparable support time (≥12 days, n = 36). Data at day 9 after starting ECMO therapy were set as “day 0”, other values depicted accordingly. Data are presented as median and interquartile range. *, p≤0.05 compared to day 0 (refers to black dots). Differences between patients with and without exchange on respective days (a, p≤0.001; b, p≤0.01; c, p≤0.05).

Progression of a device-related coagulation disorder was the reason for an elective system-exchange in the remaining 32 patients, which were divided into two groups:

Eight patients presented with a device-induced hyperfibrinolysis. As shown in Figure 4, the FG concentration decreased significantly below the normal value of 200 mg/dl, accompanied by an increase in DD levels within four days before system-exchange. This was in contrast to patients (n = 36) without system-exchange after a comparable support time. The platelet count decreased, but without reaching significance. The patients in this group showed a diffuse, otherwise unaccountable bleeding tendency. After exchange, coagulation normalized and bleeding diathesis was reduced. Thus, a device-related hyperfibrinolysis was reversible by a system-exchange. Gas exchange of respective MOs was only minimally affected (data not shown).

**Figure 5:**
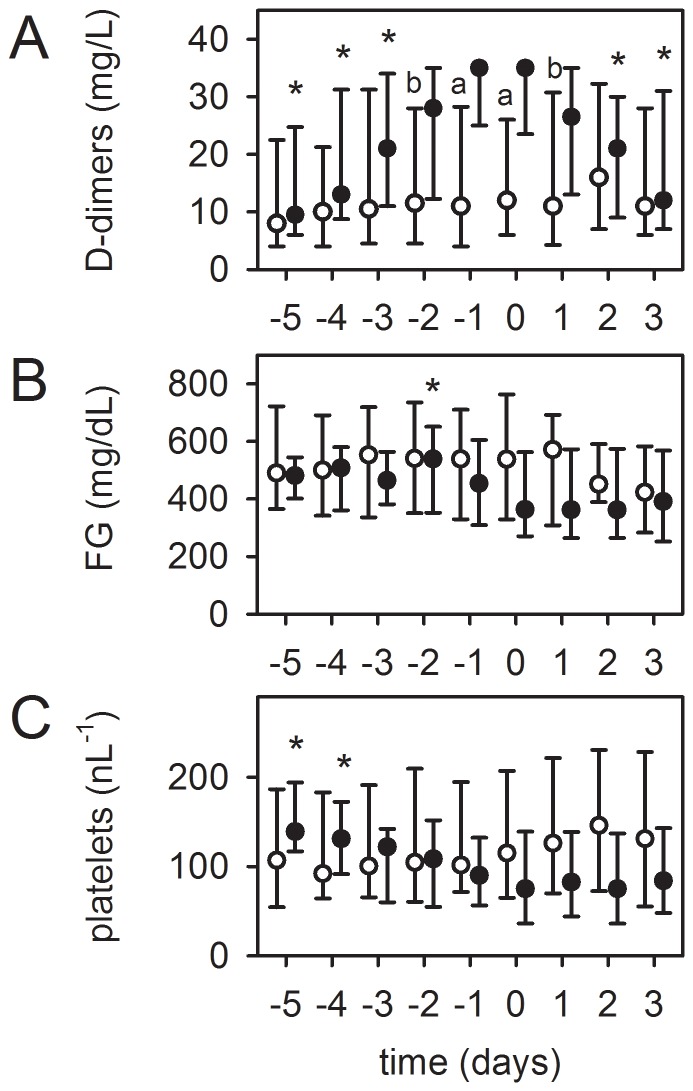
Clot formation and local fibrinolysis in the MO as a reason for an elective system-exchange. Timeline of respective values before and after sytem-exchange, “day 0”  =  system-exchange. In 24 patients (black dots), the MO was exchanged after a significant increase in D-dimer levels (A) without relevant alteration of fibrinogen (FG) levels (B). At the same time, platelet count decreased (C). After exchange, D-dimer concentration decreased and platelet counts remained stable. Circles: Patient population without exchange and comparable support time (≥12 days, n = 36). Data at day 9 after starting ECMO therapy were set as “day 0”, other values depicted accordingly. Data are presented as median and interquartile range. *, p≤0.05 compared to day 0 (refers to black dots). Differences between patients with and without exchange on respective days (a, p≤0.001; b, p≤0.01).

The remaining 24 patients had a mainly local fibrinolysis in the MO due to progressive clot formation. As shown in Figure 5, these also presented with a significant increase in DD levels and a decrease in platelet counts (not significant) within 3 to 4 days before system-exchange, as opposed to patients without system-exchange after a comparable support time. However, the FG concentration remained unchanged, although 40% of these patients developed bleeding complications. Furthermore, in seven patients gas transfer capability worsened before exchange (40% decrease in pO_2 post_MO [n = 3] and higher levels of pCO_2 post_MO [38–43 mmHg] despite high gas flow rates [6–10 l/min]). In 5 MOs the dpMO increased moderately (1.8 [1.4–2.2] -fold, p = 0.001). In one patient, frHb levels were slightly increased. Exchange of the MO reversed the changes in DDs and platelets, improved gas transfer capability of the MO, reduced frHb levels and normalized dpMO (2.0 [1.6–2.4] -fold decrease, p = 0.018).

### Technical complications of different ECMO systems

An overview of technical complications for the different systems is shown in Table 5. Due to large differences in sample size, statistical analysis was not performed and conclusions have to be drawn cautiously. The PLS-system was used most frequently (42%) as it was the first system commercially available. Technical complications leading to a system-exchange were seen in about 30% of the ECMO treatments and were independent of the ECMO system used. Mechanical failure was a rare complication and mainly seen in PLS- and HL-systems. The CH- and ECC-systems were exchanged in about 60% of cases due to acute clot formation in the pump head or acute oxygenator thrombosis, whereas the HL-system was exchanged electively in 79%. Support with an HL-system showed more ECMO-induced coagulation disorders. WGT of the MO was only documented in 12% (n = 10) of the exchanged systems, and 60% were related to the PLS-MOs. Pump head thrombosis (n = 13) was documented for the PLS-system (n = 5, 14%), CH-system (n = 5, 28%), ECC-system (n = 2, 22%) and for the iLA-activve system (n = 1, 17%).

**Table 5:** Overview of technical complications for the different ECMO systems.

ECMO systems	All	PLS	CH	HL	ECC	Others
Type of membrane		PMP	PMP	PMP	PMP	PMP
Biocompatible coating		Heparin (Bioline)	Heparin (Bioline)	Heparin (Rheoparin)	Phosphoryl-cholin	Heparin
MO exchange-surface (m^2^)		1.8	1.8	1.9	1.2	
Heat exchanger material, surface (m^2^)		Polyurethan 0.6	Polyurethan 0.6	Polyurethan 0.45	Steel 0.14	
Type of blood pump		Centrifugal (Rotaflow)	Centrifugal integrated (Rotassist)	Diagonal (Deltastream DP3)	Centrifugal integrated (Revolution)	
All patients, n (% of all)	265	112 (42)	51 (19)	47 (18)	36 (14)	4 (2)^b^, 15 (6)^a^
Patients with exchange, n (% of all patients)	83 (31)	36 (32)	18 (35)	14 (30)	9 (25)	1 (25) b, 5 (30)^ a^
**Acute exchange**	**37 (45)**	**15 (42)**	**11 (61)**	**3 (21)**	**5 (56)**	**3 (50)**
**Mechanical failure**	**8 (10)**	**5 (14)**	**0 (0)**	**2 (14)**	**0 (0)**	**1 (17)^a^**
Cannula	1 (1)	0 (0)	0 (0)	0 (0)	0 (0)	1 (17)
Pump	5 (6)	4 (11)	0 (0)	1 (7)	0 (0)	0 (0)
MO	2 (2)	1 (3)	0 (0)	1 (7)	0 (0)	0 (0)
**Acute clot formation**	**29 (35)**	**10 (28)**	**11 (61)**	**1 (7)**	**5 (55,5)**	**2 (33)**
Pump head	13 (16)	5 (14)	5 (28)	0 (0)	2 (22,2)	1 (17)^a^
MO	16 (19)	5 (14)	6 (33)	1 (7)	3 (33,3)	1 (17)^a^
**Elective exchange**	**46 (55)**	**21 (58)**	**7 (39)**	**11 (79)**	**4 (44)**	**3 (50)**
**Progressive clot formation**	**42 (50)**	**19 (53)**	**7 (39)**	**9 (64)**	**4 (44)**	**3 (50)**
Worsening of gas transfer	10 (12)	6 (17)	1 (6)	1 (7)	0 (0)	2 (33)^a+b^
Device-related coagulation disorder	32 (39)	13 (36)	6 (33)	8 (57)	4 (44)	1 (17)^a^
**Suspected infection**	**4 (5)**	**2 (5)**	**0 (0)**	**2 (14)**	**0 (0)**	**0 (0)**

PLS  =  PLS-System and CH  =  Cardiohelp-System, Maquet Cardiopulmonary, Hirrlingen, Germany. HL  =  Hilite7000LT oxygenator-System, Medos Medizintechnik, Stolberg, Germany. ECC  =  ECC.05 system, Sorin Group, Modena, Italy. Others: ^a^iLA-activve-system, Novalung, Heilbronn, Germany;

b Quadrox-ID Pediatric oxygenator + Rotaflow pump, Maquet Cardiopulmonary, Hirrlingen, Germany. PMP  =  Polymethylpenten. Data are presented as n (%).

## Discussion

**Figure 6:**
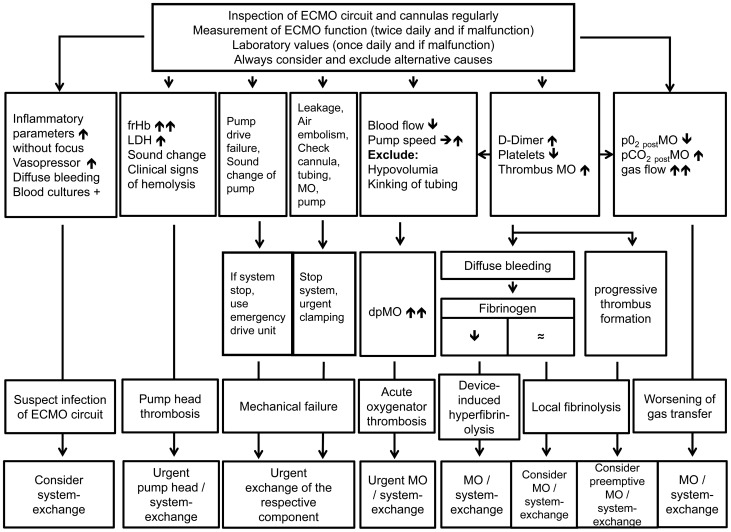
Flowchart for detecting and managing technical complications on ECMO. Not all predictors have to be present. Differential diagnoses always have to be ruled out before pathologic values can be attributed to a failing ECMO system. Different types of failure can appear together and the decision for making an exchange is often multifactorial. Therefore, usually irrespective of the leading cause of technical failure, a complete system-exchange is done, as seen in this study. If respective components are available and only one single technical failure is present, it is also possible to exchange only the failing component.

The most common technical complication with ECMO is clot formation [20]. This retrospective review detailing technical reasons for an ECMO system-exchange in 83/265 patients showed that life-threatening acute mechanical ECMO failure occurred in 10%, and an acute thrombosis of the oxygenator or blood pump in 35%, of which more than half of the cases showed preceeding alterations in gas exchange, laboratory values or an increased resistance of the MO. 55% of the system-exchanges could be done electively due to worsening of gas transfer or device-related coagulation disorders. Daily monitoring of gas exchange and pressure drop in the MO, as well as coagulation and hemolysis parameters allowed identification of developing complications in the majority of cases (see Figure 1 and Figure 6). Consequently, the number of acute system-exchanges can be decreased and the imminent system-exchanges could be performed during regular working hours and not on an emergency basis. Infection of the circuit seems to be a rare complication, but should be considered when elevated inflammatory parameters are observed, or the patient develops unaccountable bleeding diathesis after long-term ECMO support.

Since the first application of ECMO in adults with ARDS [21], types and rates of complications occurring on ECMO have influenced the outcome of these critically ill patients [9]. The incidence of various mechanical complications during ECMO support in adults can generally be classified into pump failure (4.7–20%), MO failure (21%) and blood clots within the system (3.2–22%) [9],[13],[22], all described in the literature as acute events. Especially events such as AOT and PHT are rarely predictable and the resultant acute emergency might endanger the ECMO patient. The substantial technological improvements of the ECMO circuits (e.g. small foreign surface, heparin coating of the entire ECMO system), as well as intensified circuit management in our ECMO center have reduced the incidence of acute complications. AOT and PHT accounted for 35% of the complications and were foreseeable in nearly 50% of the cases. Nevertheless, technical complications occurred that cannot be foreseen (such as leakage of the MO or failure of the blood pumps), but remained infrequent events (10%). There were no circuit or tubing ruptures. An electrical or mechanical pump drive dysfunction was seen in 3 patients and was bridged by a hand crank. Only in one patient was a rupture of the membrane of a dual-lumen Avalon cannula caused by intense head movements of the patient seen, resulting in an air embolism in the ECMO circuit.

Two of four patients receiving activated coagulation factor seven while on ECMO needed an acute system-exchange due to oxygenator thrombosis. Thus, activated coagulation factor seven on ECMO should only be given for severe live-threatening intractable bleeding.

Neither elective nor acute system-exchange affected the mortality of our patients. In subgroup analyses, patients with a system-exchange compared to those without spent longer time on ECMO, which was probably the primary reason for the need for a system-exchange. A higher incidence of acute renal failure during extracorporeal support seen in these patients was probably also caused by the longer duration of their critical illness.

An elective system-exchange was often multifactorial, but mainly due to a device-related coagulation disorder and worsening gas exchange efficiency of the MO. Device-related coagulation disorders, which account for 50% of the system-exchanges in our cohort, can be divided into mainly local fibrinolysis due to clot formation in the MO and device-induced hyperfibrinolysis. Both are often accompanied by a drop in platelet count, worsening of gas exchange and a diffuse bleeding tendency, all of which can be reversed by a system-exchange. Local fibinolysis is a weak criterion for an oxygenator exchange. However, in 29% of cases it is associated with worsening of gas transfer of the MO, in 40% with a diffuse bleeding tendency, and can progress to a device-induced hyperfibrinolysis associated with bleeding complications often requiring the substitution of blood products. This puts the patient at risk to transfusion-associated side effects and generates costs which have to be weighed against the costs and risks of a system-exchange, since the system-exchange can reverse the device-related coagulation disorder. Furthermore, since a device-related coagulation disorder is the main reason for a system-exchange, and the rate of transfused blood products is quite low in our ECMO cohort, an earlier exchange might reduce bleeding, the need for transfusions/substitution of blood products and thus reduce patient risk and save resources. However, the increased efficacy of this strategy must be proven in a prospective trial.

Worsening of gas transfer led to a system-exchange in ten patients. Gas transfer inside the MO is a complex process [23],[24], and CO_2_ removal is more efficient than oxygenation due to the higher solubility and faster diffusion properties of CO_2_ [25]. Although a minimum rate of fresh gas flow is necessary to oxygenate blood sufficiently, increasing the gas flow rate further will not lead to substantial improvement in oxygenation, but merely reduce PaCO_2_ [1]. Worsened gas exchange led to a significant increase in gas flow over time. In our experience, an increase in gas flow over 12 l/min did not improve gas transfer. An increase in blood flow might also improve O_2_ transfer and CO_2_ removal. However, an increase in blood flow rate is also accompanied by an increased risk of mechanical blood cell trauma. In our experience, a decrease in pO_2 post_MO of more than 50% compared to the initial value, and an elevation of pCO_2 post_MO above 40 mmHg at high gas flow rates (≥10 l/min) are threshold values that indicate worsened gas transfer capability and demand an elective system-exchange unless ECMO weaning within a short time is possible. If the gas transfer is only moderately reduced, it is possible to use the MO for prolonged periods of time with some fibrin/clots in the circuit. Worsening of gas transfer as the main exchange criterion accounted for only 12% of the system-exchanges, but it often accompanied other reasons for system-exchange. Hence the criteria for an elective exchange were most often multifactorial, e.g. coagulation disorder and worsening of gas transfer capability, plus increase in ventilatory aggressiveness, with ECMO weaning being considered impossible within the next several days.

Since fibrin deposition and clot formation are the main reasons for worsening of gas transfer, early detection might help prevent sudden loss of MO function. Visual control of the MO and pump head is limited because the clots are often localized in non-accessible parts of the MO. However, multidetector computed tomography (MDCT) allows the detection of thrombotic deposits in the non-visible regions of the MO [26]. Nevertheless, such technology is not yet clinically established. This study showed that monitoring of gas transfer data (gas flow, blood flow, O_2_ and CO_2_ transfer capacity), the pressure drop across the MO and of DD might facilitate early identification of respective diffusion barriers.

Colonization of the gas exchange membrane with gram-positive bacteria such as enterococci [19] or staphylococci [18] is an indication for a system-exchange. However, verification of suspected infection while on ECMO is difficult. Even in cases of infection/colonization of foreign material, blood cultures often remain negative while on antibiotic therapy. In this study, only four patients with septic diseases received a new system due to suspicion of microbiological colonization that could be proven in two cases after disassembly of the MOs. In the other two patients the suspicion rose clinically. A rise in inflammatory parameters without clinical source and an unaccountable diffuse bleeding diathesis might be induced by a system/MO infection and might trigger a system-exchange. Overall, microbial contamination of the ECMO circuit remained an infrequent event in our ECMO center, as the insertion and nursing of the insertion sites is done under strict sterile conditions, and the ECMO circuit is, except for dialysis, not used for other purposes (e.g. administration of i.v. medications).

There were no important differences regarding the technical complications between the different ECMO systems, albeit absolute numbers were low and conclusions have to be drawn cautiously.

To our knowledge, this is the first study that reviewed gas transfer data, MO pressures, coagulation and hemolysis parameters over time and their relevance for predicting an ECMO system-exchange (Table 6, Figure 6). Finally, the decision to exchange the ECMO system was routinely based on the combination of the above-mentioned laboratory parameters, ventilator parameters, worsening of gastransfer and increase in dpMO. Over the last 5 years, the number of system-exchanges per year remained unchanged (25–30%), of which about half of the exchanges were based on acute events.

**Table 6:** Summary of technical ECMO failure and diagnosis.

Type of failure	Monitoring Parameter	Acceptable value on ECMO	Value suggesting malfunction
Oxygenator thrombosis	Visual inspection	No/minimal clot formation	Pronounced/subtotal clotting
	dpMO (mmHg)/ECMO blood flow (l/min)	Device dependent [15]	30–50% increase in dpMO
	**DDimers**	<10 mg/l	>25–30 mg/l
	platelets	>50/nl	Continuous or rapid decrease
Device-related coagulation disorder	Fibrinogen	>200 mg/dl	<200 mg/dl
Gas exchange failure	PO_2_ post MO	>400 mmHg	<200 mmHg
	PCO_2_ post MO (with 10–12 l/min sweep gas flow)	<30 mmHg	>40–45 mmHg or PCO_2_ pre MO – PCO_2_ post MO<5 mmHg
Clots in pumphead	LDH	<350 U/l	Rapid and substantial increase (>1000 U/l)
	**Free Hb**	<50–100 mg/l	Rapid and substantial increase (>300 mg/l)
	*potentially platelets*	*>50/nl*	*rapid decrease*
	Sound of the pump	Normal silent noise	Abnormal noise/vibration of pump head

Type of ECMO failure and predictors with acceptable values during ECMO and values suggesting failure. These are not solely pathognomonic and not all predictors have to be present in specific types of ECMO failure. Differential diagnoses always have to be ruled out before pathologic values can be attributed to a failing ECMO system. Different types of failure can appear together and a decision for a system-exchange is often multifactorial.

Limitations of this study include its retrospective character, the subsumption of data from six different ECMO systems, and being based only on a single-center experience report. Unfortunately, the collection of a control group with only acute system-exchanges failed.

## Conclusions

Technical complications remain critical during ECMO therapy and occured in about 30% of the ECMO treatments. 45% of these system-exchanges had to be done urgently, whereas the remaining could be done electively. In 10% unpredictable mechanical complications, and in 5% a suspected infection of the device as a rare complication were the reasons for a system-exchange. Clot formation in the MO with worsening of gas transfer and device-related coagulation disorder, as well as clot formation in the pump head leading to hemolysis accounted for 85% of the technical complications. Control of the gas exchange capability and pressure drop across the MO, as well as monitoring of coagulation and hemolysis parameters over time allow for earlier identification of these complications and can reduce emergency exchanges.

### Key messages

Unpredictable acute mechanical ECMO failure occurred in 10% of the system-exchanges.

Daily monitoring of gas transfer and pressure drop of the MO, coagulation (DD, FG, platelets) and hemolysis (frHb, LDH) parameters allow for earlier identification of developing complications, which can decrease the need for emergency system-exchanges.

Device-related coagulation disorder can be reversed by a system-exchange.

Infection of the circuit seems to be an infrequent complication.

## Supporting Information

File S1 **Coagulation, hemolysis, inflammation, anticoagulation, ventilation and gas exchange parameters in all patients with and without system-exchange.** Description of data: Timeline of coagulatory parameters (AT III in %, aPTT in sec., aPT in %, D-dimers in mg/l, Fibrinogen (FG) in mg/dl, Platelet count/nl), inflammatory parameters (Leukocyte count/nl, C-reactive protein (CRP) in mg/l), hemolysis parameters (free Hemoglobin (frHb) in mg/l, Lactatdehydrogenase (LDH) in U/l), Noradrenalin consumption in ug/min/kg bodyweight, Heparin administration in U/h/kg bodyweight, ventilatory parameters (minute ventilation in l/min, respiratory rate in breaths/min, tidal volume (TV) in ml, positive endexspiratory pressure (PEEP) in cmH_2_O, peak inspiratory pressure (Pmax) in cmH_2_O, fraction of inspired oxygen (FiO_2_) and arterial blood gas values (PaO_2_/FiO_2_ in mmHg, hemoglobin in g/dl, PaO_2_ in mmHg, PaCO_2_ in mmHG, apH) in all patients with and without system-exchange. Data are presented as median and interquartile range.
(PDF)

File S2 **Coagulation, hemolysis, inflammation and anticoagulation before acute or elective system-exchange.** Titel of data: Description of data: Timeline of coagulatory parameters (AT III in %, aPTT in sec., aPT in %, D-dimers in mg/l, Fibrinogen (FG) in mg/dl, Platelet count/nl), inflammatory parameters (Leukocyte count/nl, C-reactive protein (CRP) in mg/l), hemolysis parameters (free Hemoglobin (frHb) in mg/l, Lactatdehydrogenase (LDH) in U/l), Noradrenalin consumption in ug/min/kg bodyweight and Heparin administration in U/h/kg bodyweight before acute and elective system-exchange. Data are presented as median and interquartile range.
(PDF)

File S3 **Settings of mechanical ventilation and arterial blood gases of the 10 patients with an elective system-exchange due to worsening of gas transfer.** Description of data: Timeline (4 days before system-exchange until 3 days after exchange) of arterial PO_2_ and PCO_2_, (both in mmHg), peak inspiratory pressure (PIP in cm H_2_O), positive endexspiratory pressure (PEEP, in cmH_2_O), tidal volume (TV, in ml), minute ventilation (in l/min) and FiO_2_ of the ventilator displayed as box-and-whisker plots (median, quartile, minimum, maximum). ★ p<0,05 compared to day of exchange (day 0).
(TIF)

File S4 **Indications and procedures.** Description of data: Indication for vv ECMO, approach to acute system-exchange, infection control and antibiotic therapy strategies.
(DOCX)
