# Supplementary figures and images for: Technical Complications during Veno-Venous Extracorporeal Membrane Oxygenation and Their Relevance Predicting a System-Exchange – Retrospective Analysis of 265 Cases
Source: PLoS One. 2014 Dec 2;9(12):e112316. doi: 10.1371/journal.pone.0112316 (PMC4251903; doi:10.1371/journal.pone.0112316)

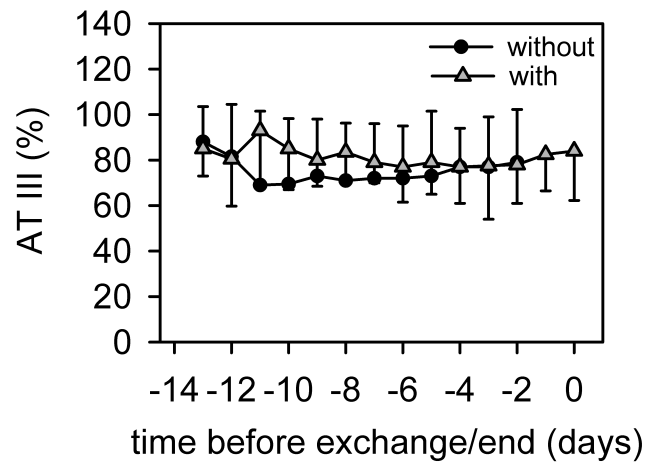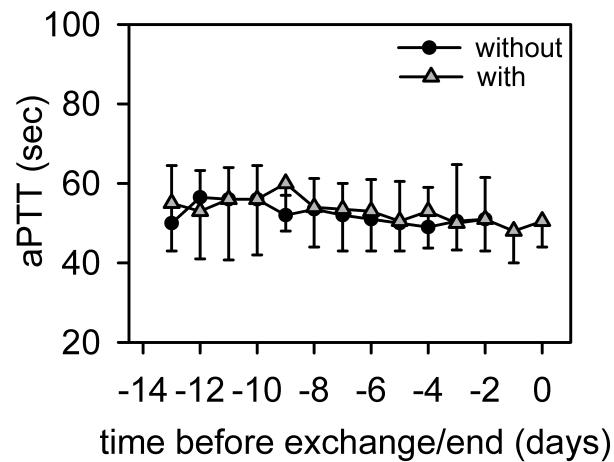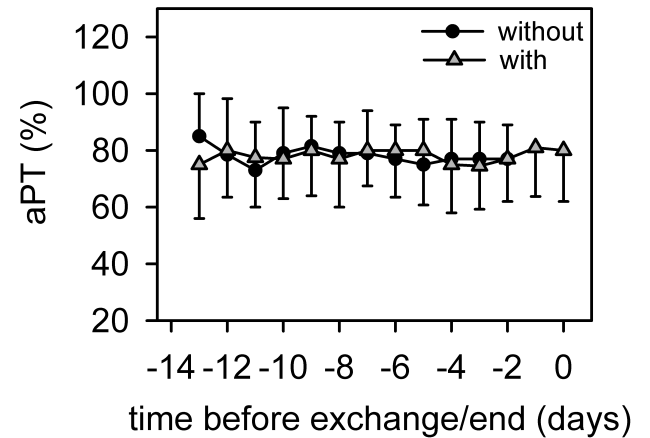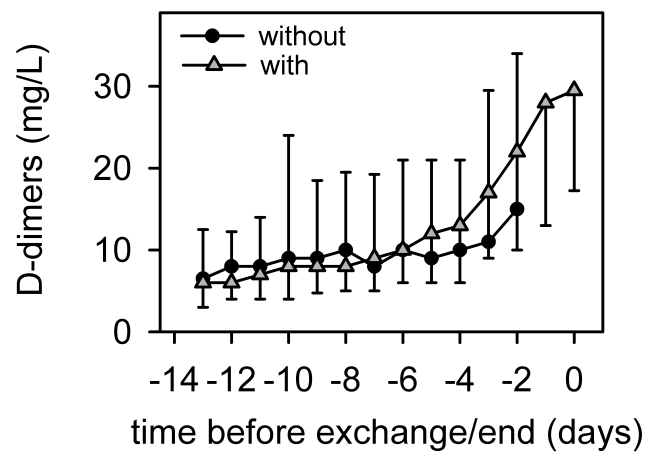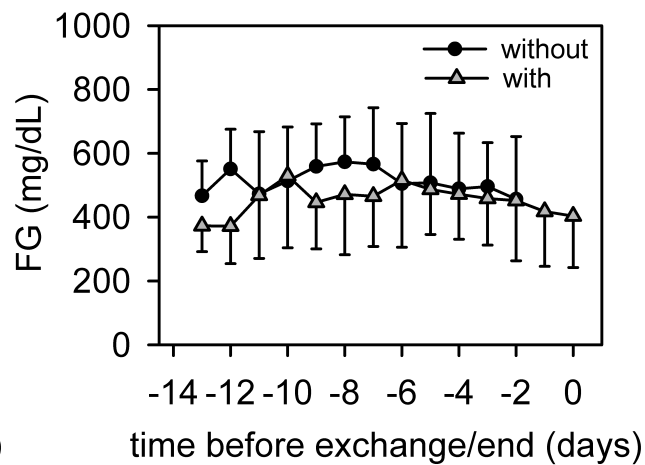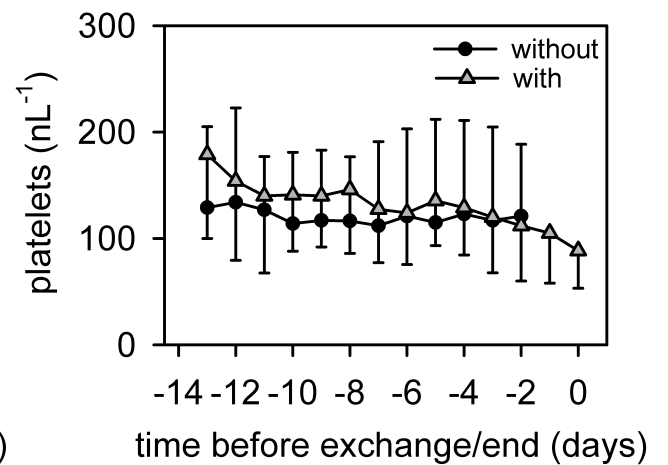

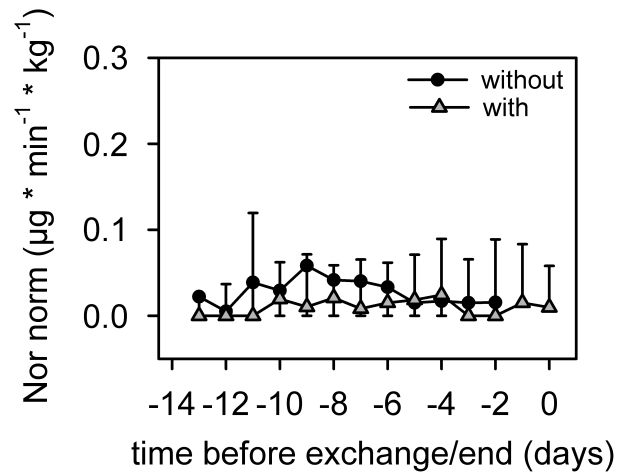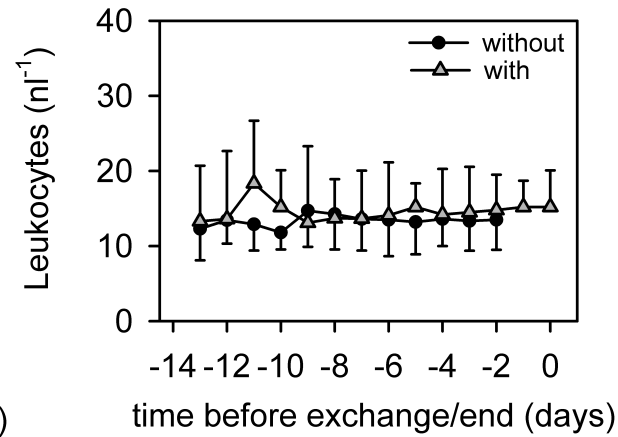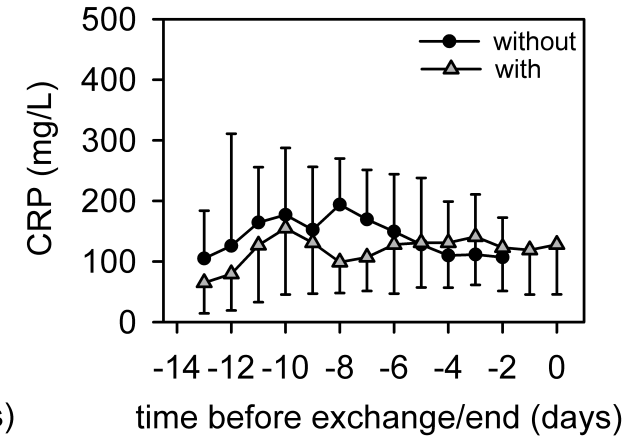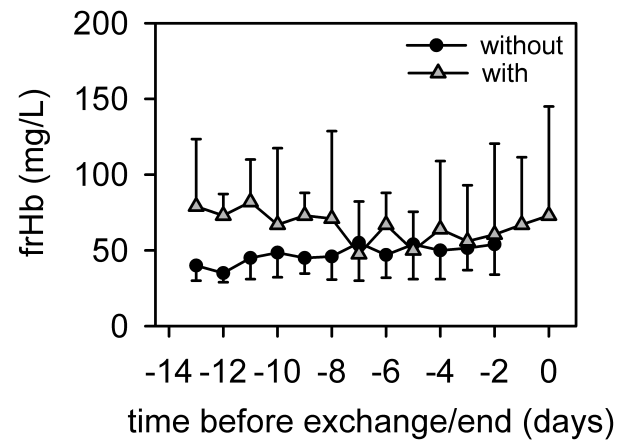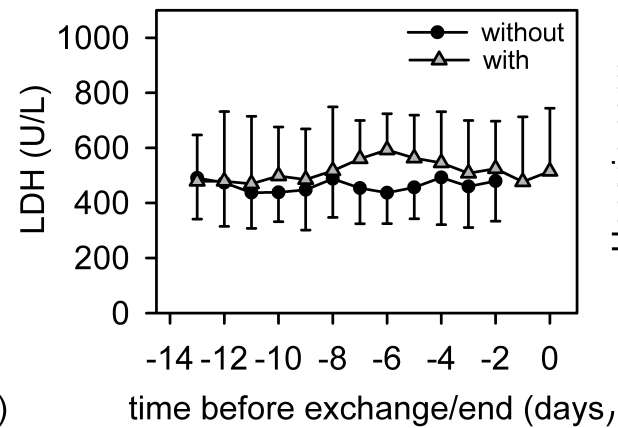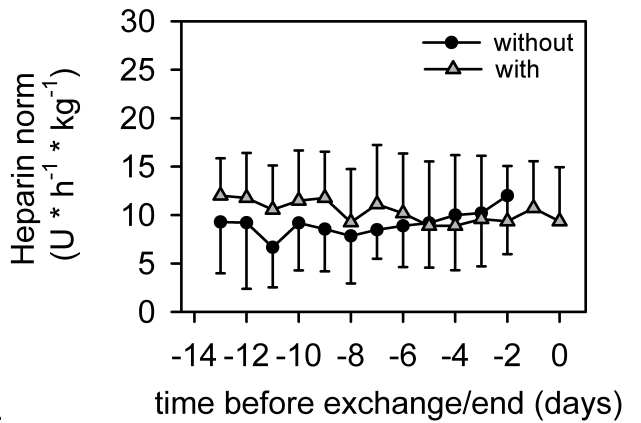

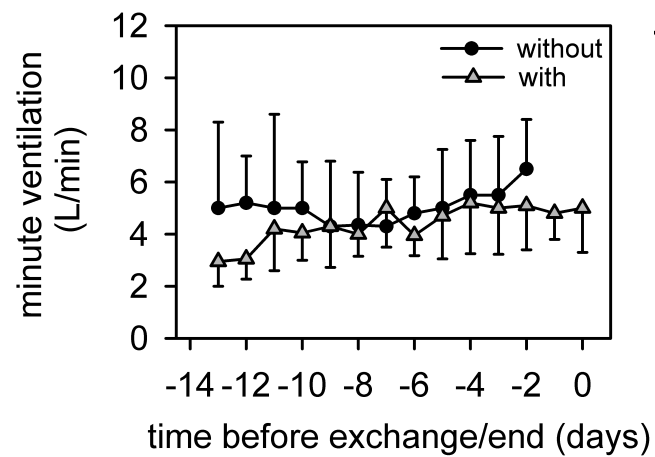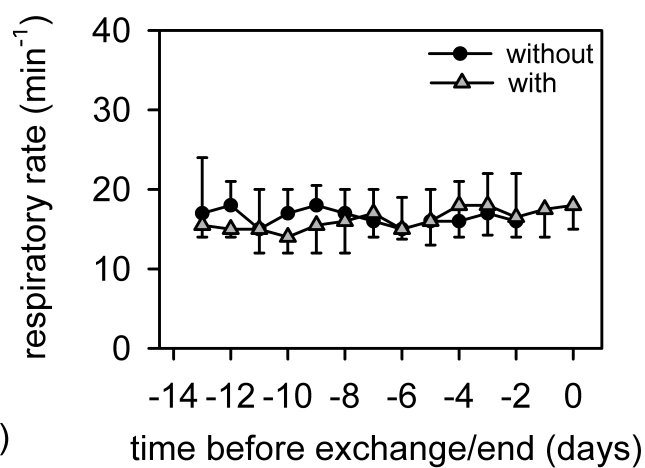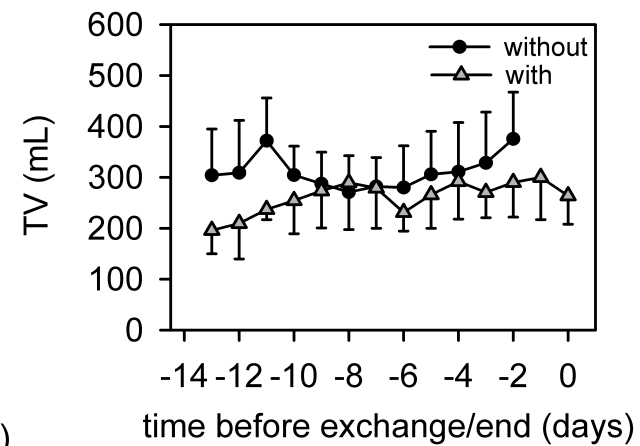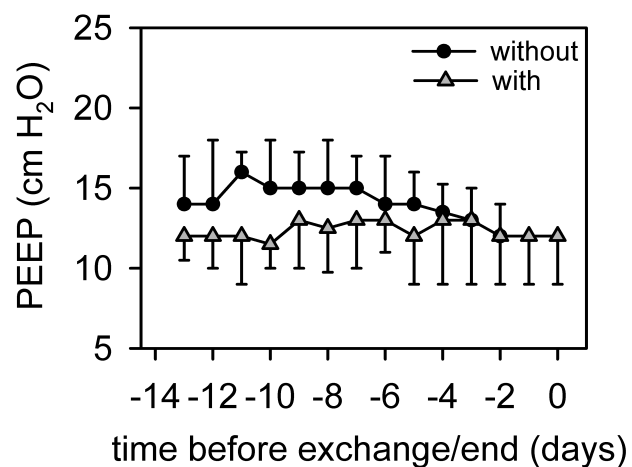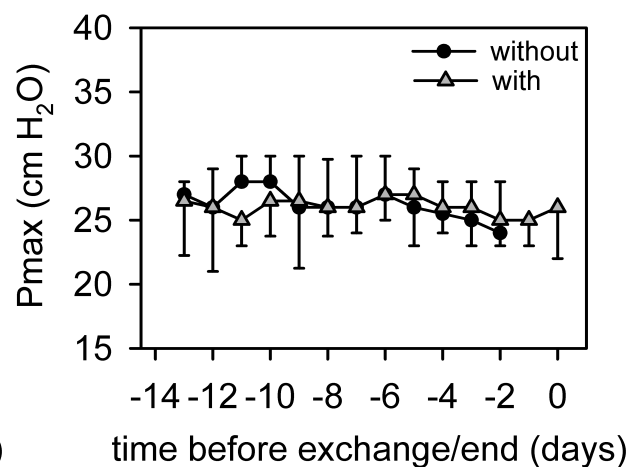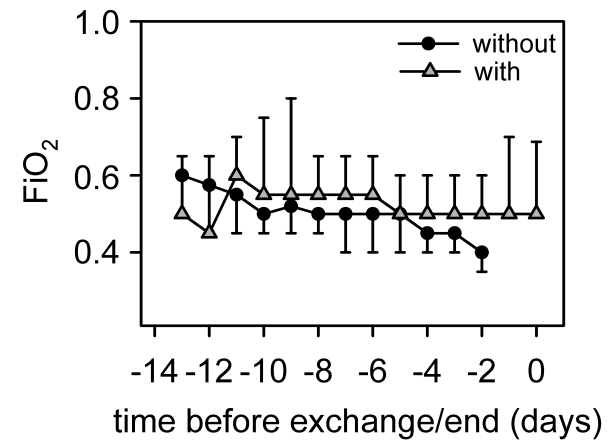

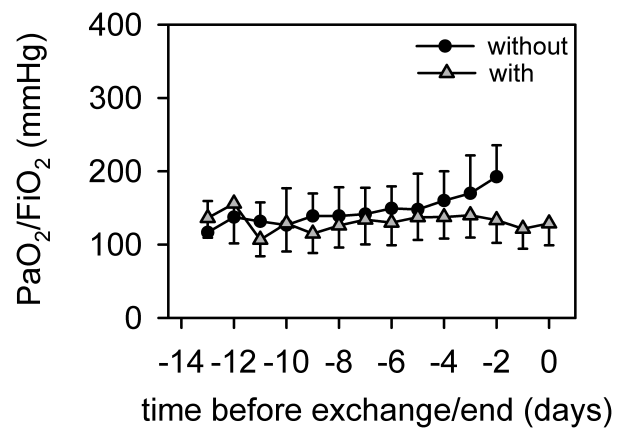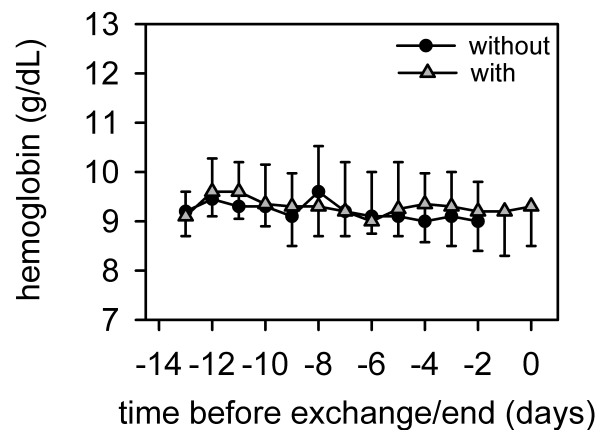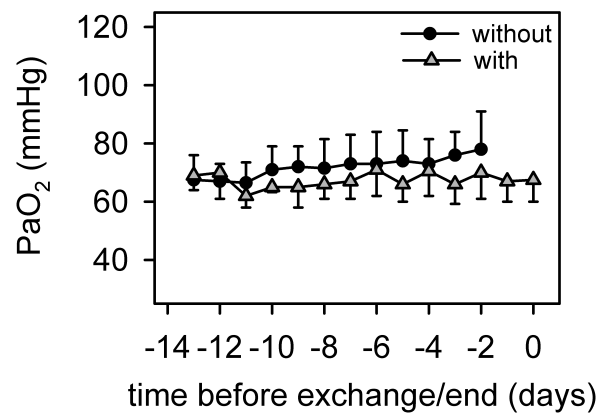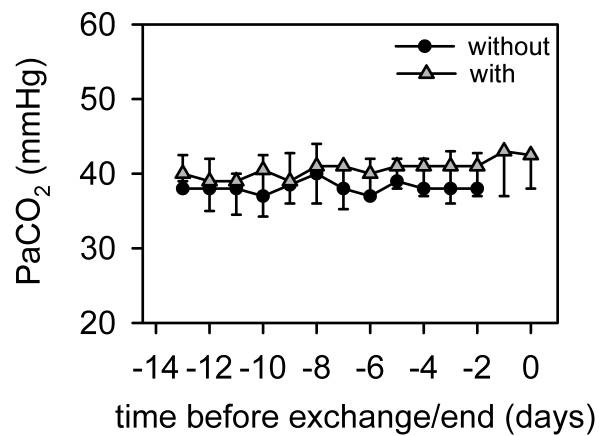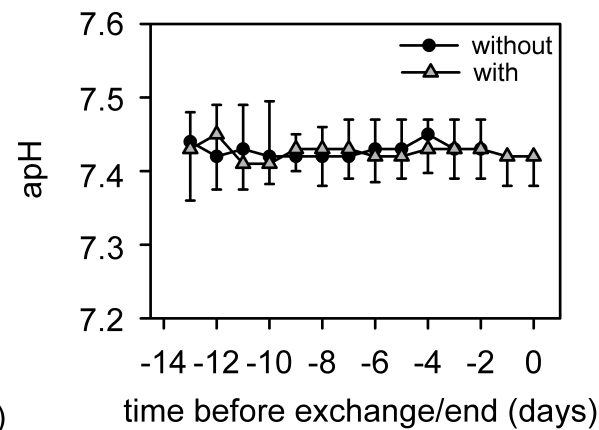

Supplement: File S1 — Coagulation, hemolysis, inflammation, anticoagulation, ventilation and gas exchange parameters in all patients with and without system-exchange. Description of data: Timeline of coagulatory parameters (AT III in %, aPTT in sec., aPT in %, D-dimers in mg/l, Fibrinogen (FG) in mg/dl, Platelet count/nl), inflammatory parameters (Leukocyte count/nl, C-reactive protein (CRP) in mg/l), hemolysis parameters (free Hemoglobin (frHb) in mg/l, Lactatdehydrogenase (LDH) in U/l), Noradrenalin consumption in ug/min/kg bodyweight, Heparin administration in U/h/kg bodyweight, ventilatory parameters (minute ventilation in l/min, respiratory rate in breaths/min, tidal volume (TV) in ml, positive endexspiratory pressure (PEEP) in cmH2O, peak inspiratory pressure (Pmax) in cmH2O, fraction of inspired oxygen (FiO2) and arterial blood gas values (PaO2/FiO2 in mmHg, hemoglobin in g/dl, PaO2 in mmHg, PaCO2 in mmHG, apH) in all patients with and without system-exchange. Data are presented as median and interquartile range. (PDF) [file pone.0112316.s001.pdf]

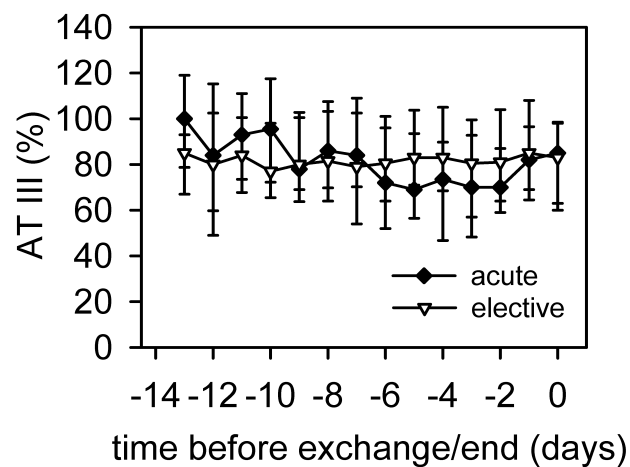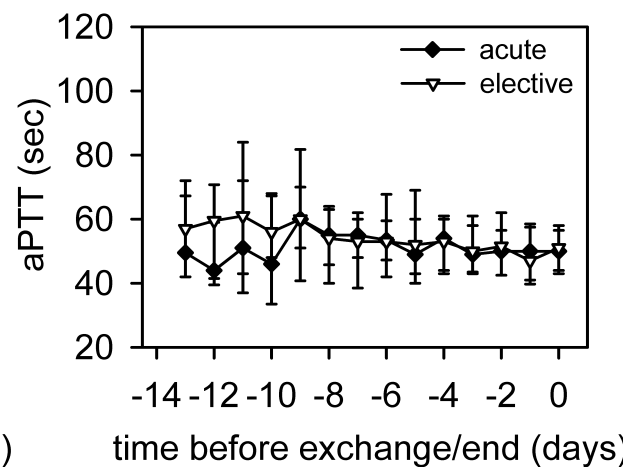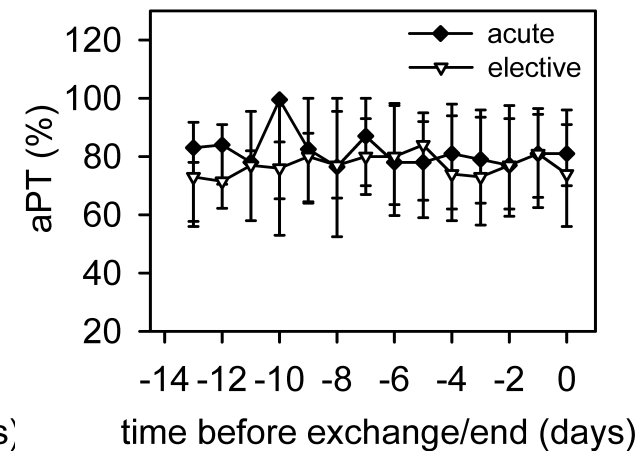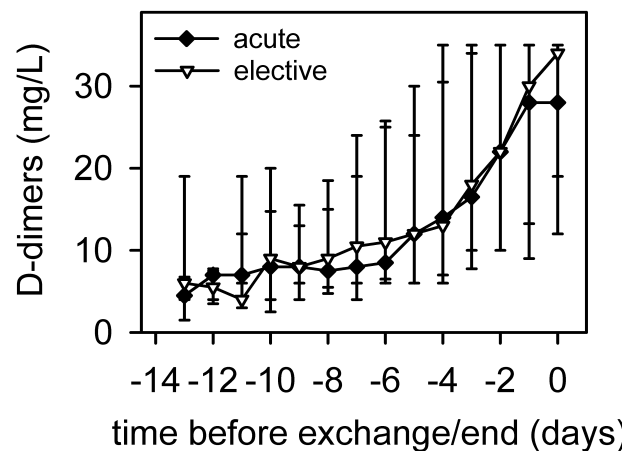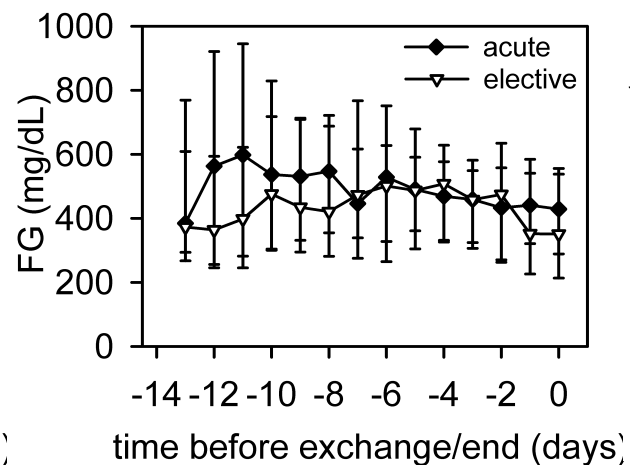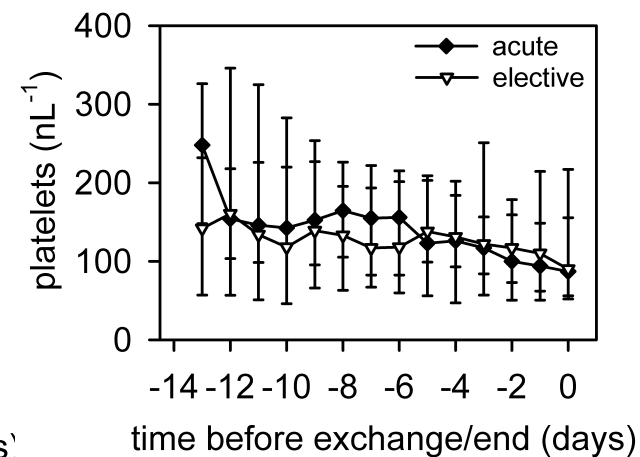

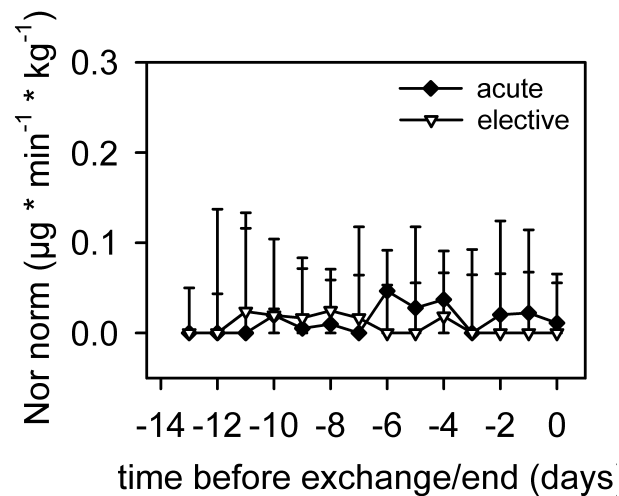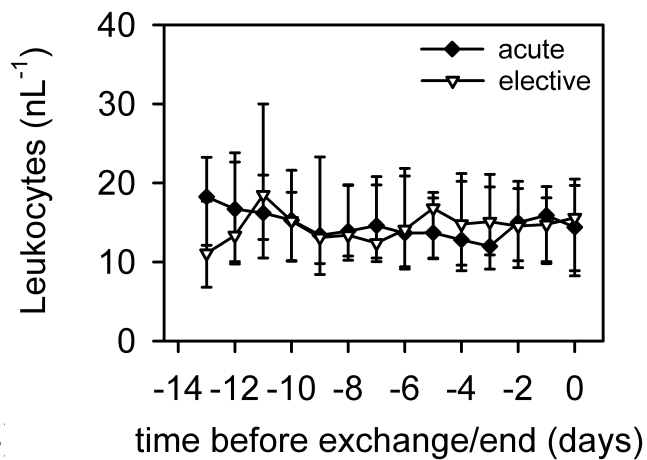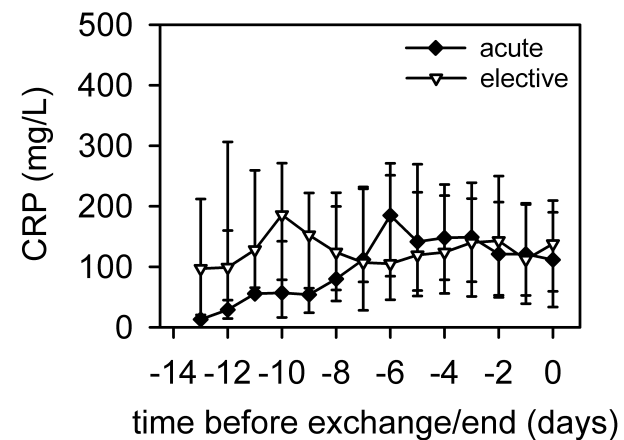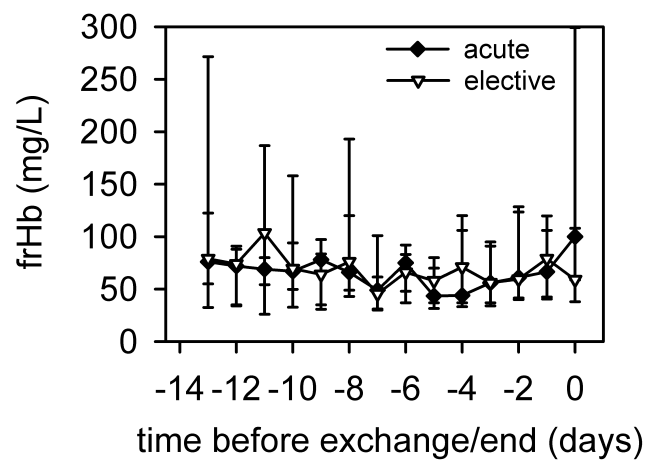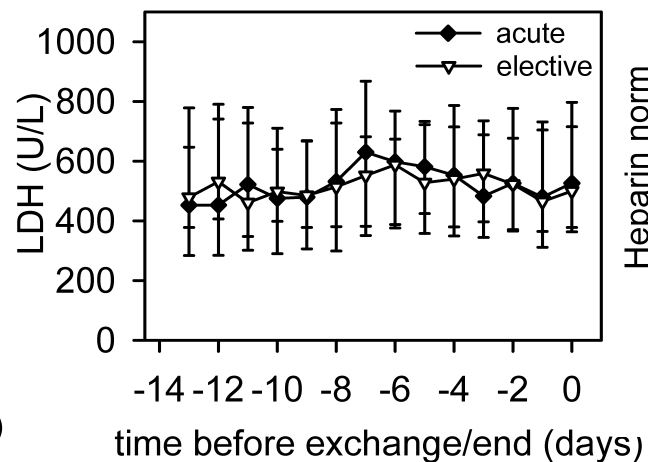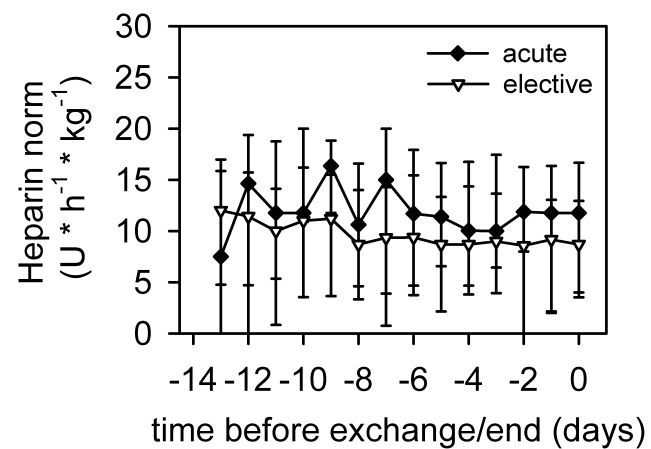

Supplement: File S2 — Coagulation, hemolysis, inflammation and anticoagulation before acute or elective system-exchange. Titel of data: Description of data: Timeline of coagulatory parameters (AT III in %, aPTT in sec., aPT in %, D-dimers in mg/l, Fibrinogen (FG) in mg/dl, Platelet count/nl), inflammatory parameters (Leukocyte count/nl, C-reactive protein (CRP) in mg/l), hemolysis parameters (free Hemoglobin (frHb) in mg/l, Lactatdehydrogenase (LDH) in U/l), Noradrenalin consumption in ug/min/kg bodyweight and Heparin administration in U/h/kg bodyweight before acute and elective system-exchange. Data are presented as median and interquartile range. (PDF) [file pone.0112316.s002.pdf]

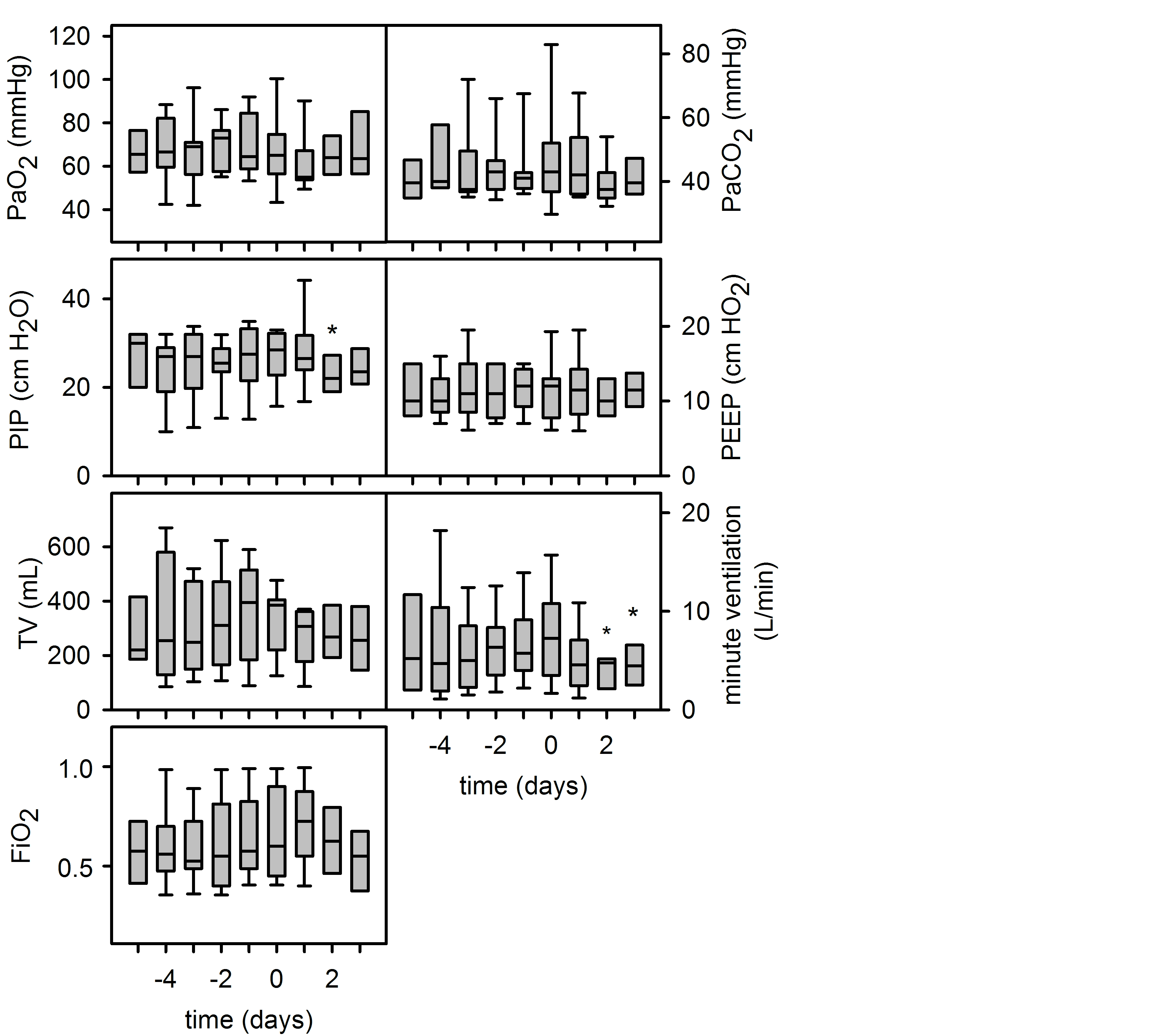

Supplement: File S3 — Settings of mechanical ventilation and arterial blood gases of the 10 patients with an elective system-exchange due to worsening of gas transfer. Description of data: Timeline (4 days before system-exchange until 3 days after exchange) of arterial PO2 and PCO2, (both in mmHg), peak inspiratory pressure (PIP in cm H2O), positive endexspiratory pressure (PEEP, in cmH2O), tidal volume (TV, in ml), minute ventilation (in l/min) and FiO2 of the ventilator displayed as box-and-whisker plots (median, quartile, minimum, maximum). ★ p<0,05 compared to day of exchange (day 0). (TIF) [file pone.0112316.s003.tif]
