# Supplementary material for: Technical Complications during Veno-Venous Extracorporeal Membrane Oxygenation and Their Relevance Predicting a System-Exchange – Retrospective Analysis of 265 Cases
Source: PLoS One. 2014 Dec 2;9(12):e112316. doi: 10.1371/journal.pone.0112316 (PMC4251903; doi:10.1371/journal.pone.0112316)
Supplement: File S4 — Indications and procedures. Description of data: Indication for vv ECMO, approach to acute system-exchange, infection control and antibiotic therapy strategies. (DOCX) [file pone.0112316.s004.docx]

**Indications for vvECMO therapy**

The indication for vvECMO was fulfilled if severe acute respiratory distress syndrome (ARDS) with a Murray lung injury score (LIS) of 3 – 4, and an arterial partial pressure of oxygen (PaO_2_) / fraction of inspired oxygen (FiO_2_) index below 80 mmHg was present despite optimization of conservative therapy. A relative indication for vvECMO was present with a Murray lung injury score of 2 – 3, and a PaO_2_/FiO_2_ index below 150 mmHg, severe respiratory acidosis (pH < 7.25), inspiratory pressure over 30 cm H_2_O or severe air leaks. Left heart failure as a cause for a pulmonary edema was excluded by echocardiography or by a wedge pressure below 18 mmHg. Optimization of gas exchange followed an institutional protocol, including initial recruitment maneuvers, prone positioning, inhalation of nitric oxide or iloprost, and high-frequency oscillatory ventilation.

**Approach to an acute MO / system-exchange**

ECMO-trained nurses and intensivists regularly control the system parameters (blood flow, pump speed and sound, gas flow, gas transfer of the ECMO, respiratory parameters and blood gas of the patient, coagulation and hemolysis parameters), and in case of an acute failure alert an ECMO pager. The pager is supervised by a perfusionist on duty around the clock. The Department of Perfusion offers technical ECMO service for all ICUs and is responsible for providing technical equipment (e.g. ready-to-use primed ECMO system). Nurses and intensivists are trained to use emergency drive units, i.e. to bridge the time until the ECMO specialist arrives at bedside. The time from appearance of severe problems to arrival of the ECMO specialist usually takes only a few minutes.

**Infection control strategy**

The infection control strategy is based on the published recommendations and national guidelines of the German Robert Koch Institute and its Division of Applied General and Hospital Hygiene (FG14), as well as its Commission for Hospital (healthcare associated) Hygiene and Infectious Disease Prevention (e.g. general preventive measures such as hand hygiene, prevention of ventilator-associated pneumonias (VAP), prevention of catheter-related infections, etc.). Adherence to these guidelines is incorporated into standard best intensive care practices, and compliance is carefully monitored by our institution's Institute of Hospital Hygiene. In particular, the following steps are taken for prevention of VAP: oral hygiene, elevation of the upper part of the body, closed endotracheal suctioning, weaning of ventilation as early as possible, etc. For the prevention of catheter-related infections, all catheters are inserted under strict sterile conditions, the changing of the respective dressings and blood is drawn under sterile conditions and catheters are changed in case of fever, rising inflammatory parameters and/or local signs of infection, etc. Accordingly, the ECMO is inserted under strict sterile conditions, as well as standard use of octenisept (Schülke & Mayr GmbH, Norderstedt, Germany) when changing respective dressings. The ECMO circuit is used solely for dialysis and not for other purposes such as the application of drugs or infusions. In case of suspected infection of the ECMO system, the ECMO circuit is changed, although the cannulas usually remain in situ unless there is an insertion site infection, which is a very rare finding.

**Monitoring of infections**

Monitoring of infections was done clinically and by measuring inflammatory parameters. In case of clinical signs of infection (e.g. shivering/fever) and/or an increase in inflammatory parameters (leucocytes, c-reative protein, procalcitonin), a search for a specific focus was undertaken (usually a CT scan of the body or an ultrasound of the abdomen and chest x-ray), with respective specimens sent for culture. Arterial and i.v. lines were changed and peripheral and central blood cultures drawn. The antibiotic therapy was escalated and later adapted according to the microbiological results. The therapeutic response was monitored by the above-mentioned inflammatory parameters. No routine screening for infections or routine blood cultures were done. Only in case of an immunocompromized patient, were weekly CMV PCR (EDTA blood) and Aspergillus antigen (serum) tests done.

**Antibiotic therapy**

Most of the patients had infectious diseases (70 – 80 % pneumonia and sepsis-related ARDS) and were on broad spectrum antibiotics. According to the focus, organ insufficiencies and unit guidelines, the initially calculated antibiotic therapy was chosen and adapted according to the microbiological results based on consultation with an infectious diseases specialist. Usually respiratory specimens (bronchial secretions, bronchoalveolar lavage), blood cultures, and urine/stool samples were sent for culture. In general, patients with pneumonia were treated with acylureido penicillins + ß-lactamase inhibitor and a macrolide or gyrase inhibitor, or in case of an ongoing infection were escalated to vancomycin, carbapenem and an antifungal agent. The antibiotic therapy was reviewed regularly by antibiotic stewardship ward rounds (microbiologist, virologist, infectious diseases specialist, pharmacist, and consultant of the ICU).
